# Internet of Samples (iSamples): Toward an interdisciplinary cyberinfrastructure for material samples

**DOI:** 10.1093/gigascience/giab028

**Published:** 2021-05-07

**Authors:** Neil Davies, John Deck, Eric C Kansa, Sarah Whitcher Kansa, John Kunze, Christopher Meyer, Thomas Orrell, Sarah Ramdeen, Rebecca Snyder, Dave Vieglais, Ramona L Walls, Kerstin Lehnert

**Affiliations:** Gump South Pacific Research Station, University of California, BP 244, Moorea 98728, French Polynesia; Berkeley Institute for Data Science, University of California, 190 Doe Library, Berkeley, CA 94720, USA; Berkeley Natural History Museums, University of California, Valley Life Sciences Bldg, 3101, Berkeley, CA 94720, USA; Open Context, The Alexandria Archive Institute, 125 El Verano Way, San Francisco, CA 94127, USA; Open Context, The Alexandria Archive Institute, 125 El Verano Way, San Francisco, CA 94127, USA; California Digital Library, University of California, Office of the President, 1111 Franklin Street, Oakland, CA 94607, USA; National Museum of Natural History, Smithsonian Institution, 10th St. & Constitution Ave. NW, Washington, D.C., 20560, USA; National Museum of Natural History, Smithsonian Institution, 10th St. & Constitution Ave. NW, Washington, D.C., 20560, USA; Lamont-Doherty Earth Observatory, Columbia University, P.O. Box 1000 61 Route 9W Palisades, NY 10964, USA; National Museum of Natural History, Smithsonian Institution, 10th St. & Constitution Ave. NW, Washington, D.C., 20560, USA; Biodiversity Institute, The University of Kansas, Dyche Hall, 1345 Jayhawk Blvd, Lawrence, KS 66045, USA; Bio5 Institute, University of Arizona, 1657 E Helen St, Tucson, AZ 85718, USA; Lamont-Doherty Earth Observatory, Columbia University, P.O. Box 1000 61 Route 9W Palisades, NY 10964, USA

**Keywords:** material sample, specimen, data standards, cyberinfrastructure, unique identifiers, persistent identifiers, collections, geoscience, bioscience, archaeology

## Abstract

Sampling the natural world and built environment underpins much of science, yet systems for managing material samples and associated (meta)data are fragmented across institutional catalogs, practices for identification, and discipline-specific (meta)data standards. The Internet of Samples (iSamples) is a standards-based collaboration to uniquely, consistently, and conveniently identify material samples, record core metadata about them, and link them to other samples, data, and research products. iSamples extends existing resources and best practices in data stewardship to render a cross-domain cyberinfrastructure that enables transdisciplinary research, discovery, and reuse of material samples in 21st century natural science.

## Background

Material samples from natural and built environments are fundamental to many branches of science and are increasingly needed for interdisciplinary research with critical societal relevance, such as sustaining natural resources, controlling infectious diseases, and coping with environmental change. Scientific collections have entered the realm of big data with the advent of simultaneous sampling across large areas and repeated sampling of the same area [1–3]. Many (perhaps most) material samples, however, are not accessioned into institutional collections but remain “hidden” in laboratories, offices, and basements, as researchers and institutions often lack the resources and expertise to properly curate them [4]. Harnessing existing sample-based data for science is cumbersome and often impractical because data about most material samples are difficult or impossible to Find, Access, Interoperate, and Reuse—they are simply not FAIR [5]. As a consequence, the full value of material samples and the data derived from them is rarely realized, either for basic scientific research or societal applications. For example, published DNA sequence data often lack the geographic metadata needed to understand the origin and spread of pathogens [6]. Maximizing the value of today's samples for tomorrow's science requires cyberinfrastructure designed to facilitate sharing and reuse across the material sample value chain and to accommodate the interdisciplinary nature of many samples (Box 1). Unleashing societal benefits from material samples requires linking them to derived data and published interpretations of those data—also essential steps to making sample-based scientific knowledge reproducible, credible, and useful. To achieve these linkages, material samples need globally unique, persistent, and resolvable identifiers with reliably accessible and trustable standards-based metadata describing the sample and its provenance. Finally, sample cyberinfrastructure must ease frictions of software (machines) interacting with the (meta)data.

Box 1: **Interdisciplinarity of Material Samples—Example from Archaeology**

**Figure 1:**
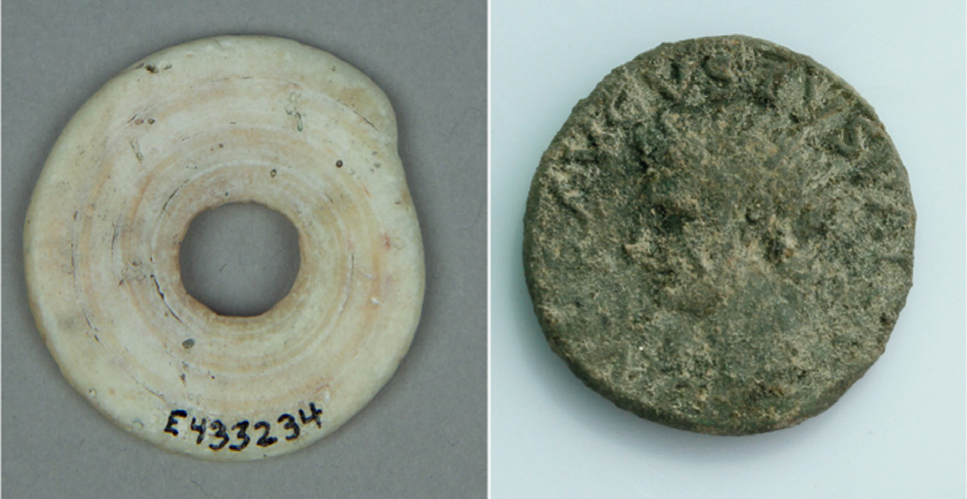
**:** Photo on left: Shell money, E433234, Department of Anthropology, Smithsonian Institution; Photo on right: Metal coin, Opitz, Mogetta, and Terrenato. Sp.Find 956, The Gabii Project: Open Context. ARK: https://n2t.net/ark:/28722/k2697cp2q.

Archaeologists study highly diverse material culture created over many millennia by peoples across the world who lived in very different regions and societies with varying cultural traditions. While material culture is difficult to describe with standard metadata, archaeologists draw upon geological and biological sources of evidence and vice versa. For example, large-scale data integration of animal remains has been used to demonstrate domestication patterns in Southwest Asia [7]. It is vital, however, that samples have appropriate provenance information and other metadata. Take the case of a research program investigating the ancient use of coins. Coins are “samples,” which should have persistent identifiers and metadata about time, space, and other aspects of archaeological context. Samples of “biological” coins, such as shell money (Figure 1, left), might also yield useful information for biologists, such as the historical biogeography of species. Similarly, samples of metal coins (Figure 1, right) have important geological aspects. Numismatists use mint marks and iconography to infer the location and date of a coin's manufacture, while geoscientists can characterize the same coin with isotope studies to allow investigation of the ore sources and post-depositional processes. iSamples will provide the cyberinfrastructure needed to facilitate such connections within and across scientific domains.

## iSamples Solution

**Figure 2:**
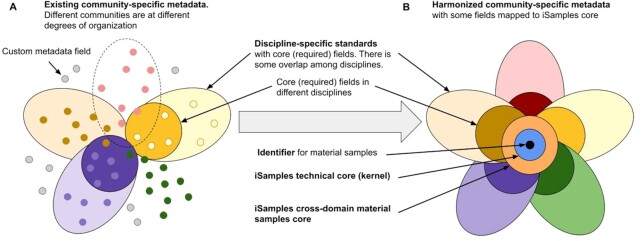
iSamples Vision: Metadata in Bloom. iSamples will extend existing domain- and sample-specific efforts, rendering a cross-domain cyberinfrastructure that can serve all samples from the natural and built environment. **(A)** Currently, each discipline creates its own community-specific metadata fields (different colored dots) and data standards (oval “petals”) based on their specialized knowledge and needs. These apply to material samples and/or a range of digital objects such as photos, datasets, genetic sequences, and publications. Disciplinary communities are at different stages of organization. The most advanced have standardized metadata fields (e.g., yellow, purple, and tan petals), sometimes with minimum required fields (darker inner petals—known as cores or kernels). Some disciplines are beginning to organize (pink dots with dotted-line petal) while others have no organization as yet (green dots). Some metadata fields cut across disciplines (the brown and green dots in the purple domain). At the cutting edge of research, new data types and custom metadata fields are constantly emerging (gray dots). **(B)** Cyberinfrastructure being built by iSamples focuses on sampling events and the resulting material samples and subsamples thereof. Metadata needed will include the material sample identifier (black dot) and its required technical core or kernel (light blue circle), as well as an iSamples cross-domain core (orange circle) that encompasses all required metadata fields shared across disciplines in the natural sciences. Promoting and facilitating community-driven metadata standards from each domain, iSamples will also support the creation of interdisciplinary metadata profiles (see Fig. 3, iSamples-in-a-Box) that include metadata fields from the iSamples core to serve the needs of interdisciplinary researchers and other users.

Recognizing the need for research infrastructure to support material samples, the U.S. National Science Foundation funded iSamples in 2020 to develop consistent services for unique and persistent sample identification and sample metadata registration across disciplines. Complementing related efforts globally, such as those of Australia's national science agency (CSIRO) and Europe's Distributed System of Scientific Collections (DiSSCo), iSamples will provide services for creating and assigning persistent, unique, and resolvable identifiers to material samples in a consistent manner across disciplines, and for registering and indexing metadata using semantic web technologies. The result will be a searchable global index of material samples linked to appropriate metadata and derived data products. iSamples aims to (i) enable previously impossible connections between diverse and disparate sample-based observations; (ii) support existing research programs and facilities that collect and manage diverse sample types; (iii) facilitate new interdisciplinary collaborations; and (iv) provide an efficient solution for FAIR samples, avoiding duplicate efforts in different domains. To achieve its goals, iSamples must incorporate and help advance diverse metadata vocabularies and standards across natural science domains (Fig. 2).

### Technical description: distributed cyberinfrastructure

**Figure 3:**
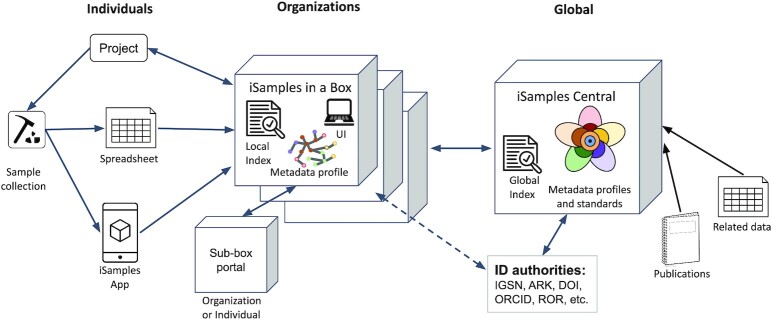
iSamples System Infrastructure. iSamples infrastructure supports individuals and organizations through 2 key components. The iSamples project will create generic code that can be used to build many instances of iSamples-in-a-Box (center). Each box is a domain or community portal that provides local services for identifier allocation and metadata collection according to metadata profiles specific to that portal. iSamples-in-a-Box would either use the existing qualifying identifiers or, in the case of non-qualifying legacy (non-unique) identifiers, generate new identifiers as needed and link them (sameAS). Individual users will push their sample metadata, collected via spreadsheets or apps (left), to the iSamples-in-a-box local index. Larger institutions may choose to create sub-boxes (e.g., a museum might create a sub-box for its field station). Boxes connect to iSamples Central (right) to verify their accounts with identifier authorities, download or synchronize metadata profiles, and—if they choose—to synchronize their metadata with the iSamples Central global index for discovery, resolution, and identifier coordination (ensuring that newly minted identifiers are associated with minimal metadata and that such records are collated locally and globally). iSamples Central manages cross-disciplinary metadata according to the model described in Fig. 2B. The iSamples Central index also stores links to related data and publications: records collated within the iSamples infrastructure are parsed to extract related objects and their predicates to determine explicit internal relations. Explicit external relations (i.e., references to entities outside of iSamples) are also collated though may be more fragile. Implicit relations are inferred by similarity of record attributes (e.g., records within a spatio-temporal region have an inferred relationship). Relations to publications requires that identifiers contained within publications be readily available, and this requires coordination with publishers to ensure extraction of the necessary information (minimally a list of identifiers occurring within a publication). Emerging infrastructure, such as the EventData service provided by Crossref, are starting to provide such capabilities on a large scale. UI: user interface.

The iSamples system has 2 core components (Fig. 3). An iSamples-in-a-Box instance is a stand-alone system that enables creation of identifiers and associated metadata, retrieval of the sample information, updates to the sample metadata (e.g., augmenting or correcting metadata or appending provenance statements), sample identifier resolution, and discovery of samples. iSamples-in-a-Box will support different scenarios. Initial use cases include (i) SESAR, which provides reliable services for sample metadata cataloguing and Global Sample Number (IGSN) registration for individual researchers and institutions [8]; (ii) GEOME, which supports capturing metadata on biological samples and links to associated genomic data [9]; and (iii) Open Context, a publishing service maintained by the Alexandria Archive Institute, which serves as a metadata repository for archaeological artefacts and ecofacts and links samples to associated data. iSamples Central is designed as a permanent Internet service that preserves and indexes sample metadata to ensure reliable discovery and retrieval. It provides a gateway between iSamples-in-a-Box instances and identifier authorities to ensure that remote iSamples-in-a-Box content is fully synchronized with the relevant authorities (e.g., IGSNs generated on iSamples-in-a-Box are synchronized with iSamples Central and the IGSN central authority). By offering services that augment existing identifier authority capabilities, iSamples Central enables support of other identifier types such as ARKs or DOIs that are not traditionally associated with material samples but are used by some organizations. iSamples Central is a central discovery and resolution service (search interface on the web and API) for any community that wishes to participate, while iSamples-in-a-Box will deliver distributed infrastructure early in the data production chain with an emphasis on the needs of specific research domains.

**Figure 4:**
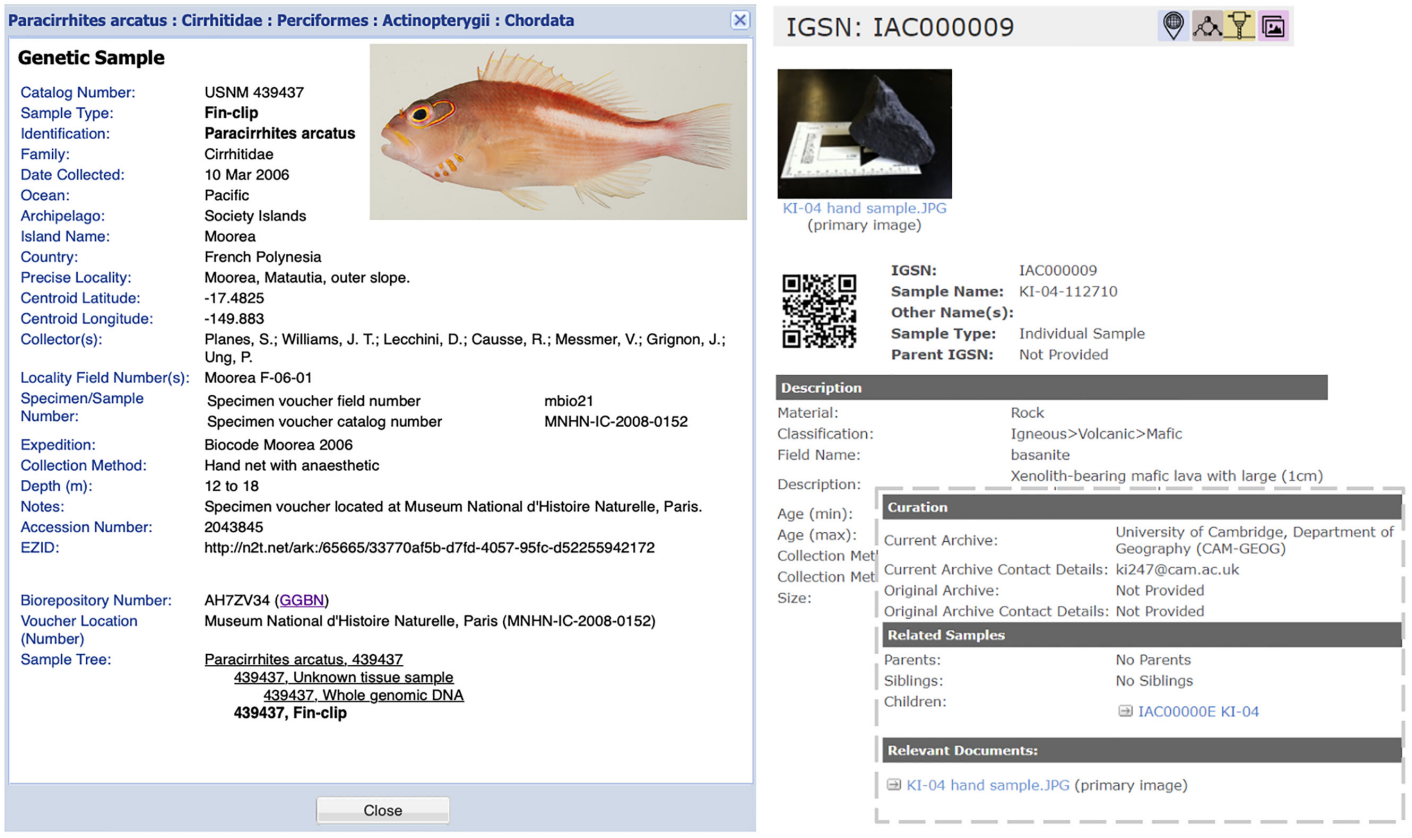
Provenance tracking—the iSample Tree. The record on the left is for a Genetic Sample in the Smithsonian's NMNH Biorepository (AG5NQ96). Each material sample has its own identifier, in this case an EZID ARK (http://n2t.net/ark:/65665/3a63356e5–953a-4666-a25f-60270f7f1dcf). The DNA was extracted from a tissue (439437, Unknown tissue sample) that was taken from a fish (*Paracirrhites arcatus*, 439437) that can be found (voucher specimen) in the National Museum of Natural History in Paris as catalog No. MNHN-IC-2008–0152. The “Sample Tree” field reveals the provenance of the DNA and also reveals another tissue sample (439437, Fin-clip) that was taken from the same fish. Clicking on any sample in the sample tree would reveal its own EZID ARK. The record on the right is for a rock sample, KI-04-112710, registered in the SESAR catalog by a research scientist. Each sample registered in SESAR is assigned an IGSN as a unique identifier, in this case IAC000009 (http://igsn.org/IAC000009). KI-04-112710 is a hand sample that is representative of primitive lavas from Antarctica and was subsequently powdered for additional analysis. In the “Related Samples” field of its profile page, the resulting rock powder is listed as a child sample, KI-04-11272010 (IAC00000E). KI-04-11272010 was resampled for phase equilibrium experiments and lists 27 children samples. The links between the records provide the provenance between the parent, child, and grandchild samples. By registering identifiers, iSamples will enable such “Sample Trees” to reveal a larger value chain, such as linking collecting events to other specimens and their derivatives and resulting data.

Provenance is often truncated in current data systems (Fig. 4). iSamples takes an event-based approach, capturing metadata upstream from Field Information Management Systems and maintaining links downstream, with metadata standards implemented or inferred at each step. Some metadata are inferred, as they must follow all parent-child relationships (e.g., “where” and “when” of the collecting event), but other types of metadata (e.g., taxonomy) cannot always be inferred. For example, a subsample from a fish might not inherit the fish's taxonomy because it might be something the fish ate or a parasite; similarly, a mineral subsampled from a rock cannot inherit the rock taxonomy.

### Sampling nature: sustainability, inclusion, and equity

While iSamples has funding to build cyber-infrastructure addressing technological barriers, significant sociological challenges must be overcome to unleash the full value of material samples. iSamples will engage scientific and technical expertise around standards and ontologies (e.g., through Research Coordination Network mechanisms). Harnessing material samples for sustainable development, however, also requires empowering a broad swath of stakeholders to benefit from material samples, related data, and research products—particularly people from whose communities the samples are derived. It is vital that standards, training materials, public outreach, and policy recommendations be equitable and inclusive. Important areas of emphasis include access and benefit sharing (Convention on Biological Diversity), Indigenous data rights, and social justice, where inequities of the past and present need to be addressed. Key steps that iSamples will pursue include the integration of Collective Benefit, Authority to Control, Responsibility, and Ethics—the “CARE principles” [10] and the adoption of Traditional Knowledge and Biocultural Labels and Notices, an initiative of “Local Contexts” that provides a mechanism for Indigenous communities to engage with cultural and research institutions to manage their traditional rights over their property and knowledge.

### Beyond iSamples

The need for permanent identifiers and robust metadata is not unique to material samples. Building a fully comprehensive Internet of Samples will require infrastructure similar to iSamples for all resources connected to samples, including datasets, images, sound recordings, and publications. iSamples will contribute to such efforts, e.g., around the concepts of “digital specimens" and networks of “extended specimens." Furthermore, while iSamples focuses on the natural sciences, material samples are important in several sectors that are increasingly interconnected, such as approaches to public health that combine ecology and medicine.

## Conclusions

iSamples will allow scientists to track natural science samples, subsamples, associated metadata, data, and research products. iSamples is a single, distributed, transdisciplinary infrastructure based on domain-neutral technologies, standards, and consistent sample identification that is extensible to accommodate domain-specific needs. iSamples aims to enhance existing research within disciplines while enabling new research across them.

## Data Availability

Not applicable.

## Abbreviations

API: Application Programming Interface; ARK: Archival Resource Key; CARE: Collective benefit, Authority to control, Responsibility, Ethics; CSIRO: Commonwealth Scientific and Industrial Research Organisation; DOI: Digital Object Identifier; FAIR: Findable, Accessible, Interoperable, Reusable; GEOME: Genomic Observatories Metadatabase; GSC: Genomic Standards Consortium; IGSN: IGSN Global Sample Number; ORCID: Open Researcher and Contributor ID; ROR: Research Organization Registry; SESAR: System for Earth Sample Registration.

## Competing Interests

The authors declare that they have no competing interests.

## Funding

This material is based upon work supported by the National Science Foundation under Grant Nos. 2004839, 2004562, 2004642, and 2004815. Any opinions, findings, and conclusions or recommendations expressed in this material are those of the authors and do not necessarily reflect the views of the National Science Foundation.

## Authors' Contributions

Much of the text is derived from the collaborative “iSamples” proposal that was submitted to the National Science Foundation following a workshop K.L. organized in August 2019 at Columbia University. N.D. put together the first draft of the present manuscript and all authors contributed to subsequent drafts, with R.L.W. adding Fig. 2, D.V. and R.L.W. Fig. 3, and C.M. Fig. 4. All authors read and approved the final manuscript.

## Supplementary Material

giab028_GIGA-D-21-00056_Original_Submission

giab028_GIGA-D-21-00056_Revision_1

giab028_Response_to_Reviewer_Comments_Original_Submission

giab028_Alex_Hardisty_Review_iSamples_04Mar2021 Alex Hardisty -- 3/4/2021 Reviewed

giab028_Reviewer_1_Report_Original_Submission Alex Hardisty -- 3/4/2021 Reviewed

giab028_Reviewer_2_Report_Original_Submission Vincent Smith -- 3/5/2021 Reviewed
