# Supplementary material for: Internet of Samples (iSamples): Toward an interdisciplinary cyberinfrastructure for material samples
Source: Gigascience. 2021 May 7;10(5):giab028. doi: 10.1093/gigascience/giab028 (PMC8103498; doi:10.1093/gigascience/giab028)
Supplement: giab028_GIGA-D-21-00056_Original_Submission [file giab028_giga-d-21-00056_original_submission.pdf]

GigaScience

Internet of Samples (iSamples): Toward an Interdisciplinary Cyberinfrastructure for Material Samples

--Manuscript Draft--

**Manuscript Number:**	GIGA-D-21-00056
**Full Title:**	Internet of Samples (iSamples): Toward an Interdisciplinary Cyberinfrastructure for Material Samples
**Article Type:**	Commentary
**Funding Information:**	National Science Foundation (2004815) — Dr David A Vieglais<br>National Science Foundation (2004642) — Dr. Neil Davies<br>National Science Foundation (2004839) — Dr Kerstin Lehnert<br>National Science Foundation (2004562) — Dr Ramona Walls
**Abstract:**	Sampling the natural world and built environment underpins much of science, yet systems for managing material samples and associated (meta)data are fragmented across institutional catalogs, practices for identification, and discipline-specific (meta)data standards. The Internet of Samples (iSamples) is a standards-based collaboration to uniquely, consistently, and conveniently identify material samples, record core metadata about them, and link them to other samples, data, and research products. iSamples extends existing resources and best practices in data stewardship to render a cross-domain cyberinfrastructure that enables transdisciplinary research, discovery, and reuse of material samples in 21st century natural history.
**Corresponding Author:**	Neil Davies, Ph.D.<br>University of California Berkeley<br>Berkeley, California UNITED STATES
**Corresponding Author Secondary Information:**	
**Corresponding Author's Institution:**	University of California Berkeley
**Corresponding Author's Secondary Institution:**	
**First Author:**	Neil Davies, Ph.D.
**First Author Secondary Information:**	
**Order of Authors:**	Neil Davies, Ph.D.<br>John Deck<br>Eric C Kansa<br>Sarah Whitcher Kansa<br>John Kunze<br>Christopher Meyer<br>Thomas Orrell<br>Sarah Ramdeen<br>Rebecca Snyder<br>David A Vieglais<br>Ramona Walls<br>Kerstin Lehnert

Powered by Editorial Manager® and ProduXion Manager® from Aries Systems Corporation

Order of Authors Secondary Information:	
Additional Information:	
Question	Response
Are you submitting this manuscript to a special series or article collection?	No
**Experimental design and statistics**<br><br>Full details of the experimental design and statistical methods used should be given in the Methods section, as detailed in our Minimum Standards Reporting Checklist. Information essential to interpreting the data presented should be made available in the figure legends.<br><br>Have you included all the information requested in your manuscript?	Yes
**Resources**<br><br>A description of all resources used, including antibodies, cell lines, animals and software tools, with enough information to allow them to be uniquely identified, should be included in the Methods section. Authors are strongly encouraged to cite Research Resource Identifiers (RRIDs) for antibodies, model organisms and tools, where possible.<br><br>Have you included the information requested as detailed in our Minimum Standards Reporting Checklist?	Yes
**Availability of data and materials**<br><br>All datasets and code on which the conclusions of the paper rely must be either included in your submission or deposited in publicly available repositories (where available and ethically appropriate), referencing such data using a unique identifier in the references and in the "Availability of Data and Materials" section of your manuscript.	Yes

Powered by Editorial Manager® and ProduXion Manager® from Aries Systems Corporation

Have you have met the above requirement as detailed in our Minimum Standards Reporting Checklist?	

Click here to access/download;Manuscript;iSamples_Marker Paper_MS.pdf

Internet of Samples (iSamples): Toward an Interdisciplinary Cyberinfrastructure for Material Samples

- Neil Davies[1,2]; ndavies@berkeley.edu; https://orcid.org/0000-0001-8085-5014
- John Deck[3]; jdeck@berkeley.edu; https://orcid.org/0000-0002-5905-1617
- Eric C. Kansa[4]; kansaeric@gmail.com; https://orcid.org/0000-0001-5620-4764
- Sarah Whitcher Kansa[4]; skansa@alexandriaarchive.org; https://orcid.org/0000-0001-7920-5321
- John Kunze[5], jak@ucop.edu; https://orcid.org/0000-0001-7604-8041
- Christopher Meyer[6], meyerc@si.edu; https://orcid.org/0000-0003-2501-7952
- Thomas Orrell[6]; orrellt@si.edu; https://orcid.org/0000-0003-1038-3028
- Sarah Ramdeen[7], sramdeen@ldeo.columbia.edu; https://orcid.org/0000-0003-1135-5942
- Rebecca Snyder[6] , snyderr@si.edu, https://orcid.org/0000-0002-0028-6139
- Dave Vieglais[8]; vieglais@ku.edu; https://orcid.org/0000-0002-6513-4996
- Ramona L. Walls[9], rwalls@email.arizona.edu; https://orcid.org/0000-0001-8815-0078
- Kerstin Lehnert[7*]; lehnert@ldeo.columbia.edu; https://orcid.org/0000-0001-7036-1977

*corresponding author

1. Gump South Pacific Research Station, University of California, BP 244 98728, Moorea, French Polynesia
2. Berkeley Institute for Data Science, University of California, Berkeley, CA 94720, USA
3. Berkeley Natural History Museums, University of California, Berkeley, CA 94720, USA
4. Open Context, The Alexandria Archive Institute, San Francisco, CA, 94127, USA
5. California Digital Library, University of California, Office of the President, Oakland, CA 94607, USA
6. National Museum of Natural History, Smithsonian Institution, Washington DC, 20560, USA
7. Lamont-Doherty Earth Observatory, Columbia University, Palisades, NY 10964, USA
8. Biodiversity Institute, The University of Kansas, KS, 66045, USA
9. Bio5 Institute, University of Arizona, Tucson, AZ 85718, USA

Abstract

Sampling the natural world and built environment underpins much of science, yet systems for managing material samples and associated (meta)data are fragmented across institutional catalogs, practices for identification, and discipline-specific (meta)data standards. The Internet of Samples (iSamples) is a standards-based collaboration to uniquely, consistently, and conveniently identify material samples, record core metadata about them, and link them to other samples, data, and research products. iSamples extends existing resources and best practices in data stewardship to render a cross-domain cyberinfrastructure that enables transdisciplinary research, discovery, and reuse of material samples in 21st century natural history.

Keywords

Material sample, specimen, data standards, cyberinfrastructure, unique identifiers, persistent identifiers, collections, geoscience, bioscience, archaeology

Background

Material samples from natural and built environments are fundamental to many branches of science and are increasingly needed for interdisciplinary research with critical societal relevance, such as sustaining natural resources, controlling infectious diseases, and coping with environmental change. Scientific collections have entered the realm of big data with the advent of simultaneous sampling across large areas and repeated sampling of the same area [1–3]. Many (perhaps most) material samples, however, are not accessioned into institutional collections but remain ‘hidden’ in labs, offices, and basements, as researchers and institutions often lack the resources and expertise to properly curate them [4]. Harnessing existing sample-based data for science is cumbersome and often impractical as data about most material samples are difficult or impossible to Find, Access, Interoperate, and Reuse -- they are simply not FAIR [5]. As a consequence, the full value of material samples and the data derived from them are rarely realized, either for basic scientific research or societal applications. For example, published DNA sequence data often lack the basic geographic metadata needed to understand the origin and spread of pathogens [6]. Maximizing the value of today’s samples for tomorrow’s science requires cyberinfrastructure designed to facilitate sharing and reuse across the material sample value chain and to accommodate the interdisciplinary nature of many samples (**Box 1**). Unleashing the societal value of material samples also requires linking them to derived data and published interpretations of those data - essential steps to making sample-based scientific knowledge reproducible, credible, and useful. In order to achieve these linkages, material samples need globally unique, persistent, and resolvable identifiers with reliably accessible and trustable standards-based metadata describing the sample and its provenance.

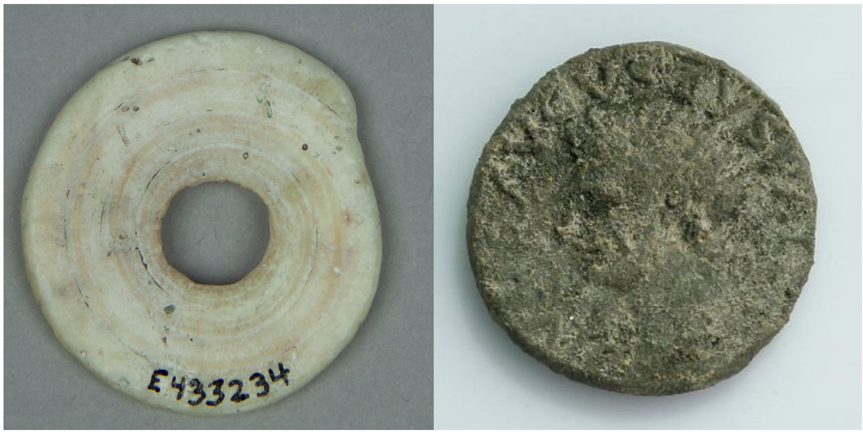

Box 1: Interdisciplinarity of Material Samples - Example from Archaeology

Archaeologists study highly diverse material culture created over many millennia by peoples across the world who lived in very different regions, societies, and cultural traditions. While material culture is difficult to describe with standard metadata, archaeologists draw upon geological and biological sources of evidence and vice versa. For example, large-scale data integration of animal remains has been used to demonstrate domestication patterns in Southwest Asia [7]. It is vital, however, that samples have appropriate provenance information and other metadata. Take the case of a research program investigating the ancient use of coins. Coins are “samples”, which should have persistent identifiers and metadata about time, space, and other aspects of archaeological context. Samples of ‘biological’ coins, such as shell money (see photo on left; E433234, Department of Anthropology, Smithsonian Institution), might also yield useful information for biologists, such as the historical biogeography of species. Similarly, samples of metal coins (see photo on right; Opitz, Mogetta, and Terrenato. Sp.Find 956, The Gabii Project: Open Context. ARK:https://n2t.net/ark:/28722/k2697cp2q) have important geological aspects. Numismatists use mint-marks and iconography to infer location and date of a coin’s manufacture, while geoscientists can characterize the same coin with isotope studies to allow investigation of the ore sources and post-depositional processes. iSamples will provide the cyberinfrastructure needed to facilitate such connections within and across scientific domains.

Main text

iSamples Solution

Recognizing the need for research infrastructure to support material samples, the U.S. National Science Foundation funded iSamples in 2020 to develop consistent services for unique and persistent sample identification and sample metadata registration across disciplines. Collaborating with similar efforts globally, iSamples will provide services for creating and assigning persistent, unique, and resolvable identifiers to material samples in a consistent manner across disciplines, and for registering and indexing metadata using semantic web technologies. The result will be a searchable global index of material samples linked to appropriate metadata and derived data products. iSamples aims to (i) enable previously impossible connections between diverse and disparate sample-based observations; (ii) support existing research programs and facilities that collect and manage diverse sample types; (iii) facilitate new interdisciplinary collaborations; and (iv) provide an efficient solution for FAIR samples, avoiding duplicate efforts in different domains. To achieve its goals, iSamples must incorporate and help advance diverse metadata vocabularies and standards across natural history domains (**Figure 1**).

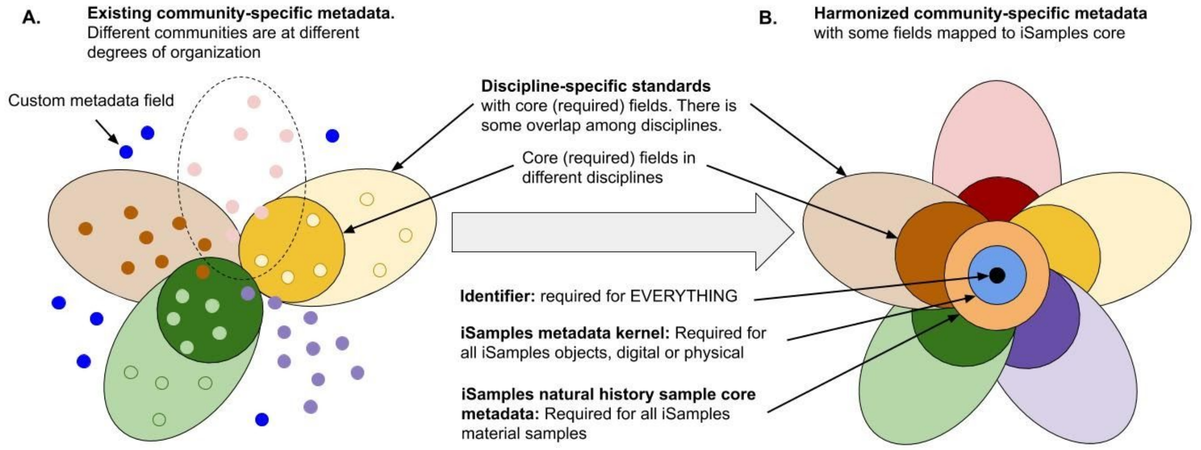

Figure 1. iSamples Vision: Metadata in Bloom

iSamples will extend existing domain- and sample-specific efforts, rendering a cross-domain cyberinfrastructure that can serve all samples from the natural and built environment. **(A)** Currently, each discipline creates its own community-specific metadata fields (different colored dots) and data standards (oval shaped 'petals') based on their specialized knowledge and needs. Disciplinary communities are at different stages of organization. The most advanced have standardized metadata fields (e.g., yellow, green, and tan petals) sometimes with minimum required fields (darker inner petals). Some disciplines are beginning to organize (pink dots with dotted-line petal) while others have no organization as yet (purple dots). Some metadata fields cut across disciplines (the brown and purple dots in the green domain). At the cutting edge of research, new data types and custom metadata fields are constantly emerging (blue dots). **(B)** Cyberinfrastructure being built by iSamples focuses on sampling events and the resulting material samples and subsamples thereof. Metadata needed will include the material sample identifier (black dot) and its required metadata kernel (light blue circle), as well as an iSamples core (orange circle) that encompasses all required metadata fields shared across disciplines in the natural history domain. Promoting and facilitating community-driven metadata standards from each domain, iSamples will also support the creation of interdisciplinary metadata profiles (see Figure 2, iSamples in a Box) that include metadata fields from the iSamples core (required) and across domains (optional) to serve the needs of interdisciplinary researchers and other users.

Technical Description: Distributed Cyberinfrastructure

The iSamples system has two core components (**Figure 2**). An **iSamples-in-a-Box** instance is a standalone system that enables creation of identifiers and associated metadata, retrieval of the sample information, updates to the sample metadata (e.g., augmenting or correcting metadata or appending provenance statements), sample identifier resolution, and discovery of samples. iSamples-in-a-Box will support different scenarios. Initial use cases include: (a) SESAR, which provides reliable services for sample metadata cataloguing and Global Sample Number (IGSN) registration for individual researchers and institutions [8]. (b) GEOME, which supports capturing metadata on biological samples, and links to associated genomic data [9], and (c) Open Context, a publishing service maintained by the Alexandria Archive Institute, which serves as a metadata repository for archaeological artefacts and ecofacts and links

samples to associated data. **iSamples Central** is designed as a permanent Internet service that preserves and indexes sample metadata to ensure reliable discovery and retrieval, and provides a gateway between iSamples-in-a-Box instances and identifier authorities to ensure that remote iSamples-in-a-Box content is fully synchronized with the relevant authorities (e.g., IGSNs generated on iSamples-in-a-Box are synchronized with iSamples Central and the IGSN central authority). By offering services that augment existing identifier authority capabilities, iSamples Central enables support of other identifier types such as ARKs or DOIs that are not traditionally associated with material samples, but are used by some organizations. iSamples Central is a central discovery and resolution service (search interface on the web and API) for any community that wishes to participate, while iSamples-in-a-Box will deliver distributed infrastructure early in the data production chain with an emphasis on the needs of specific research domains.

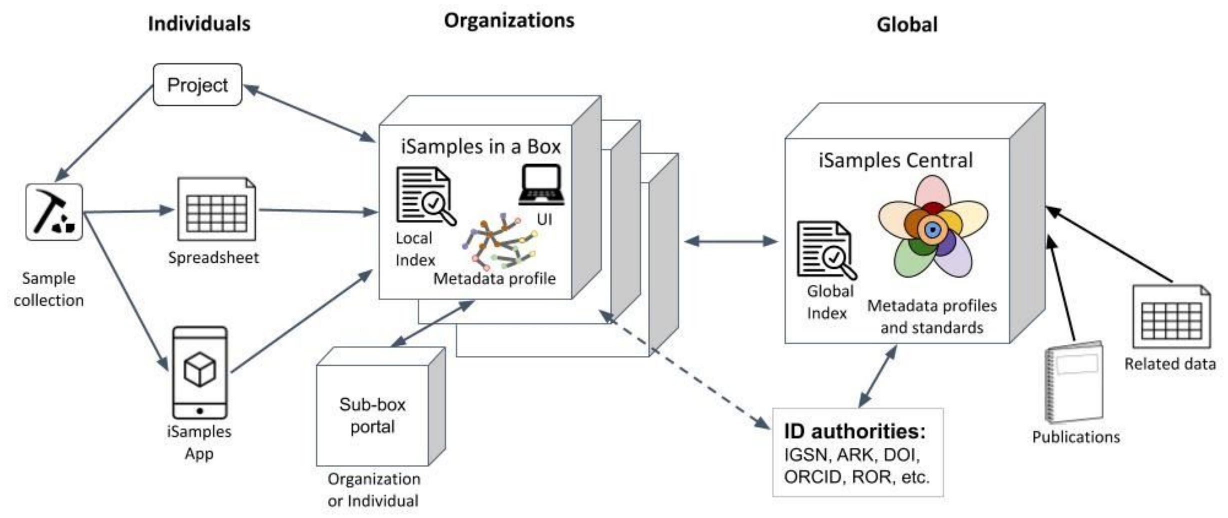

Figure 2. iSamples System Infrastructure

iSamples infrastructure supports individuals and organizations through two key components. The iSamples project will create generic code that can be used to build many instances of **iSamples-in-a-Box** (center). Each box is a domain or community portal that provides local services for identifier allocation and metadata collection according to metadata profiles specific to that portal. Individual users will push their sample metadata, collected via spreadsheets or apps (left), to the iSamples-in-a-box local index. Larger institutions may choose to create sub-boxes (e.g., a museum might create a sub-box for its field station). Boxes connect to **iSamples Central** (right) to verify their accounts with ID authorities, download or sync metadata profiles, and -- if they choose -- to sync their metadata with the iSamples Central global index for discovery, resolution, and identifier coordination. iSamples Central manages cross-disciplinary metadata according to the model described in Figure 1B. The iSamples Central index also stores links to related data and publications.

Provenance is often truncated in current data systems (**Figure 3**), iSamples takes an event-based approach capturing metadata upstream from Field Information Management Systems and maintaining links downstream, with metadata standards implemented or inferred at each step. Some metadata are inferred, as they must follow all parent-child relationships (e.g., 'where' and 'when' of the event), but other types of metadata (e.g., taxonomy) cannot always be inferred (e.g., a subsample from a fish might not inherit the fish's taxonomy as it

might be something the fish ate or a parasite; similarly, a mineral subsampled from a rock cannot inherit the rock taxonomy).

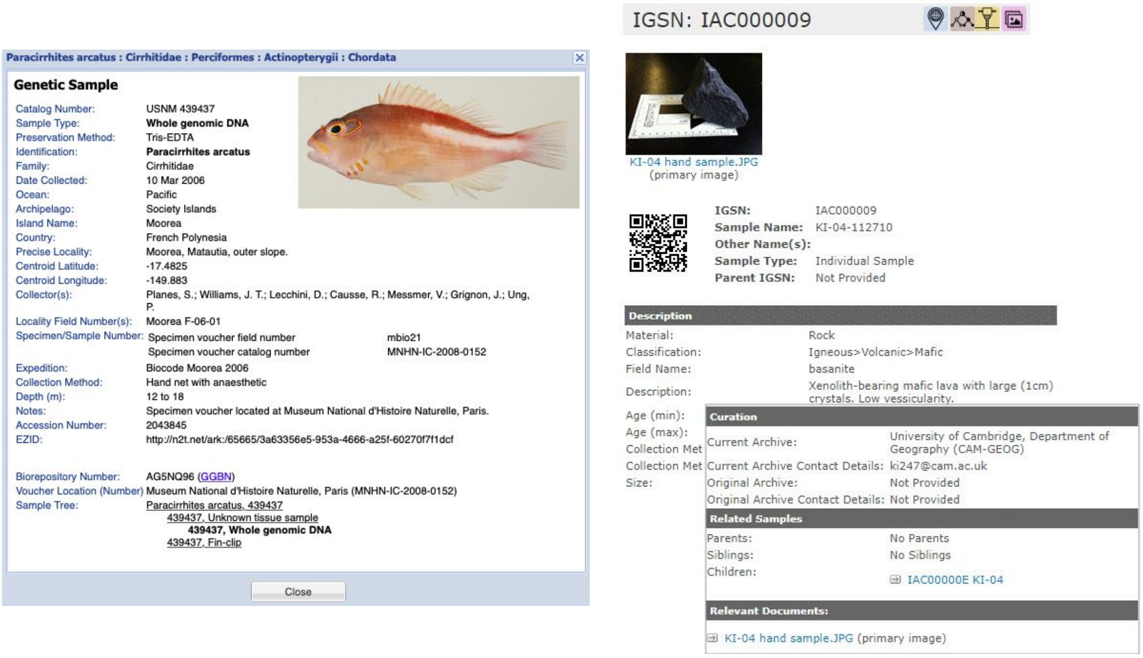

Figure 3. Provenance tracking - the iSample Tree.

The record on the left is for a Genetic Sample in the Smithsonian's NMNH Biorepository (AG5NQ96). Each material sample has its own identifier, in this case an EZID ARK, that is bolded in the relational sample tree. The DNA was extracted from a tissue (439437, Unknown tissue sample) that was taken from a fish (Paracirrhites arcatus, 439437) that can be found (voucher specimen) in the National Museum of Natural History in Paris as catalog number MNHN-IC-2008-0152. The "Sample Tree" field reveals the provenance of the DNA, and also reveals another tissue sample (439437, Fin-clip) that was taken from the same fish.

The record on the right is for a rock sample, KI-04-112710, registered in the SESAR catalog by a research scientist. Each sample registered in SESAR is assigned an IGSN as a unique identifier, in this case IAC000009. KI-04-112710 is a hand sample that is representative of primitive lavas from Antarctica and was subsequently powdered for additional analysis. In the "Related Samples" field of it's profile page, the resulting rock powder is listed as a child sample, KI-04-11272010 (IAC00000E). KI-04-11272010 was resampled for phase equilibrium experiments, and lists 27 children samples. The links between the records provides the provenance between the parent, child and grandchildren samples.

By registering identifiers, iSamples will enable such "Sample Trees" to reveal a larger value-chain, such as linking the collecting event to other specimens and their derivatives and resulting data (e.g., GenBank submissions, images).

Sampling Nature: Sustainability, Inclusion, and Equity

While iSamples has funding to build cyber-infrastructure addressing technological barriers, significant sociological challenges remain to unleashing the full value of material samples. Harnessing material samples for sustainable development, for example, requires empowering a broad swath of stakeholders to benefit from material samples, related data, and research products, particularly people from whose communities the samples are derived. It is vital that standards, training materials, public outreach, and policy recommendations are equitable and inclusive. This is particularly important in the areas of Indigenous data rights and social justice, where inequities of the past and present need to be addressed. Integration of CARE as well as FAIR principles, for example, and the adoption of tools such as Traditional Knowledge and Biocultural Labels (an initiative of “Local Contexts”) represent important steps that iSamples will pursue.

Beyond Natural History

iSamples will focus on the natural history sector – any sample where geolocation is of primary importance – but can scale to other domains. Material samples are important in a number of sectors that are increasingly interconnected, such as ecology and medicine in ‘One Health’ [10]. The need for permanent identifiers and robust metadata is not unique to material samples. Building a fully-comprehensive internet of samples will require infrastructure similar to iSamples for all resources connected to samples, including datasets, images, sound recordings, and publications.

Conclusions

iSamples will allow scientists to track natural history samples, subsamples, associated metadata, data, and research products. iSamples is a single, distributed, transdisciplinary infrastructure based on domain-neutral technologies, standards, and consistent sample identification that is extensible to accommodate domain-specific needs. iSamples aims to enhance existing research within disciplines while enabling new research across them.

List of abbreviations

ARK: Archival Resource Key
CARE: Collective benefit, Authority to control, Responsibility, Ethics
DataOne: Data Observation Network for Earth
DOI: Digital Object Identifier
FAIR: findable, accessible, interoperable, reusable
GEOME: Genomic Observatories Metadatabase
GSC: Genomic Standards Consortium
IGSN: IGSN Global Sample Number
ORCID: Open Researcher and Contributor ID

ROR: Research Organization Registry
SESAR: System for Earth Sample Registration

Declarations

Data availability

Not applicable.

Consent for publication

Not applicable.

Competing interests

The authors declare that they have no competing interests.

Funding

This material is based upon work supported by the National Science Foundation under Grant Numbers 2004839, 2004562, 2004642, and 2004815. Any opinions, findings, and conclusions or recommendations expressed in this material are those of the author(s) and do not necessarily reflect the views of the National Science Foundation.

Authors' contributions

Much of the text is derived from the collaborative “iSamples” proposal that was submitted to the National Science Foundation following a workshop KL organized in August 2019 at Columbia University. ND put together the first draft of the current manuscript and all authors contributed to subsequent drafts, with RW adding Figure 1, DV and RW Figure 2, and CM Figure 3. All authors read and approved the final manuscript.

Acknowledgements

We are grateful for input on the draft manuscript from Hong Cui and Stephen Richard, and to Leslie Weyborn for inspiration for our metadata model and Figure 1.

References

1. Davies N, Field D, Amaral-Zettler L, Clark MS, Deck J, Drummond A, et al.. The founding charter of the Genomic Observatories Network. *Gigascience*. 3:22014;

2. Buttigieg PL, Janssen F, Macklin J, Pitz K. The Global Omics Observatory Network: Shaping standards for long-term molecular observation. *Biodiversity Information Science and Standards*. Pensoft Publishers; 2019; doi: 10.3897/biss.3.36712.

3. Mirtl M, T Borer E, Djukic I, Forsius M, Haubold H, Hugo W, et al.. Genesis, goals and achievements of Long-Term Ecological Research at the global scale: A critical review of ILTER and future directions. *Sci Total Environ*. 626:1439–622018;

4. McNutt M, Lehnert K, Hanson B, Nosek BA, Ellison AM, King JL. Liberating field science samples and data. *Science*. American Association for the Advancement of Science; 351:1024–62016;

5. Wilkinson MD, Dumontier M, Aalbersberg IJJ, Appleton G, Axton M, Baak A, et al.. The FAIR Guiding Principles for scientific data management and stewardship. *Sci Data*. 3:1600182016;

6. Schriml LM, Chuvochina M, Davies N, Eloe-Fadrosh EA, Finn RD, Hugenholtz P, et al.. COVID-19 pandemic reveals the peril of ignoring metadata standards. *Scientific Data*. 7:1882020;

7. Arbuckle BS, Kansa SW, Kansa E, Orton D, Çakırlar C, Gourichon L, et al.. Data sharing reveals complexity in the westward spread of domestic animals across Neolithic Turkey. *PLoS One*. 9:e998452014;

8. Ramdeen S, Lehnert K, Markey K, Devendran S, Johansson A, Song L. Citations for physical samples: IGSN and the System for Earth Sample Registration. p. IN12B – 01.

9. Deck J, Gaither MR, Ewing R, Bird CE, Davies N, Meyer C, et al.. The Genomic Observatories Metadatabase (GeOMe): A new repository for field and sampling event metadata associated with genetic samples. *PLoS Biol*. 15:e20029252017;

10. Amuasi JH, Lucas T, Horton R, Winkler AS. Reconnecting for our future: The Lancet One Health Commission. *Lancet*. 395:1469–712020;
