# Supplementary material for: Internet of Samples (iSamples): Toward an interdisciplinary cyberinfrastructure for material samples
Source: Gigascience. 2021 May 7;10(5):giab028. doi: 10.1093/gigascience/giab028 (PMC8103498; doi:10.1093/gigascience/giab028)
Supplement: giab028_GIGA-D-21-00056_Revision_1 [file giab028_giga-d-21-00056_revision_1.pdf]

GigaScience

Internet of Samples (iSamples): Toward an Interdisciplinary Cyberinfrastructure for Material Samples

--Manuscript Draft--

**Manuscript Number:**	GIGA-D-21-00056R1
**Full Title:**	Internet of Samples (iSamples): Toward an Interdisciplinary Cyberinfrastructure for Material Samples
**Article Type:**	Commentary
**Funding Information:**	National Science Foundation (2004642) — Dr. Neil Davies<br>National Science Foundation (2004839) — Dr Kerstin Lehnert<br>National Science Foundation (2004562) — Dr Ramona Walls<br>National Science Foundation (2004815) — Dr David A Vieglais
**Abstract:**	Sampling the natural world and built environment underpins much of science, yet systems for managing material samples and associated (meta)data are fragmented across institutional catalogs, practices for identification, and discipline-specific (meta)data standards. The Internet of Samples (iSamples) is a standards-based collaboration to uniquely, consistently, and conveniently identify material samples, record core metadata about them, and link them to other samples, data, and research products. iSamples extends existing resources and best practices in data stewardship to render a cross-domain cyberinfrastructure that enables transdisciplinary research, discovery, and reuse of material samples in 21st century natural science.
**Corresponding Author:**	Neil Davies, Ph.D.<br>University of California Berkeley<br>Berkeley, California UNITED STATES
**Corresponding Author Secondary Information:**	
**Corresponding Author's Institution:**	University of California Berkeley
**Corresponding Author's Secondary Institution:**	
**First Author:**	Neil Davies, Ph.D.
**First Author Secondary Information:**	
**Order of Authors:**	Neil Davies, Ph.D.<br>John Deck<br>Eric C Kansa<br>Sarah Whitcher Kansa<br>John Kunze<br>Christopher Meyer<br>Thomas Orrell<br>Sarah Ramdeen<br>Rebecca Snyder<br>David A Vieglais<br>Ramona Walls<br>Kerstin Lehnert

Powered by Editorial Manager® and ProduXion Manager® from Aries Systems Corporation

**Order of Authors Secondary Information:**	
**Response to Reviewers:**	We are grateful to both reviewers for their careful consideration of this manuscript and below we provide point-by-point responses (bullet points) to their comments (in italics):<br><br>Reviewer #1:<br><br>Figure 3. … the explanation of the provenance (sample tree) relations among the things is correct but the assertions that the numbers in the sample tree are ARKs is incorrect. This explanation needs to be revised.<br><br>Figure 3: we have corrected the problems identified with the numbers in the sample tree<br><br>In figure 1 a distinction is made between iSamples digital objects and iSamples physical objects but no explanation is given of the difference. What is meant by the former?<br><br>We intend to eventually extend iSamples to digital objects, and the ID will be required for these as well. However, this paper simply describes our work for material samples, so we have simplified the language in figure 1 and its legend.<br><br>Also in figure 1, it is mentioned that facilitating community-driven metadata standards and adopting interdisciplinary metadata profiles will take place. These are social challenges. Later in the article, there is an admission that significant social challenges exist but not much is said about how the project aims to tackle these.<br><br>We intend to address these challenges through a Research Coordination Network "Sampling Nature". We have not cited this RCN directly, however, because the award is not yet finalized. Instead, we have revised the text to mention the importance of RCNs as a mechanism for engaging the community in the social aspects of cyberinfrastructure.<br><br>In the explanation of figure 2 the phrase 'identifier coordination' is used. What is meant by this?<br><br>Identifier coordination involves ensuring that newly minted identifiers are associated with minimal metadata and that such records are collated locally and globally. We have added this additional explanation to the Figure 2 caption.<br><br>It is said that iSamples Central index also stores links to related data and publications as well as the metadata about the samples. How these links will be captured and created is not explained.<br><br>We have added an explanation to the Figure 2 legend.<br><br>No mention is made… organization-specific (legacy) identifier types arising from historic collections ...How can these cases of non-unique identifiers be accommodated by iSamples?<br><br>We have updated the text in the Figure 2 legend to address this issue (briefly). A fuller response: While legacy non-unique identifier types are an important issue, iSamples is focused on the need for all new samples to follow best-practice in assigning unique identifiers and thus not to contribute to the scale of the problem going forward. Having said that, assurance of legacy record uniqueness can be achieved through namespacing (e.g., prefixing with an ARK shoulder) or minting of a new identifier. We plan to take advantage of the California Digital Library N2T (name to thing) resolver service to help manage legacy identifiers with an option to convert legacy identifiers into globally unique "compact identifiers". [https://doi.org/10.1038/sdata.2018.95] In either case, the original identifier is retained with a "sameAs" relation to ensure ongoing resolution, albeit with potentially multiple matches that require additional refinement. iSamples in-a-box services would allow legacy institutions without identifier best practices to participate.

Powered by Editorial Manager® and ProduXion Manager® from Aries Systems Corporation

...how will machine/software-oriented rather than human-oriented processing of digital data about samples be catered for. The article does not discuss machine-actionability of sample data at all; although the authors acknowledge that further work beyond the present project will be required.

We very much agree that machine-actionability is a key issue and iSamples represents a first step by making sample data FAIRer, and as the reviewer points out, the FAIR principles are themselves aimed at machines. We added a sentence recognizing that sample cyberinfrastructure must strive to ease frictions of software (machines) interacting with the (meta)data.

Access and benefit sharing should be mentioned alongside CARE.

We have made this addition in the text

In the text headed ‘iSamples solution’: The authors say iSamples will be ‘collaborating with similar efforts globally’ but they do not state/reference what these efforts are. The reader cannot infer what is meant here. Are similar efforts for identifying and indexing samples meant or is it similar efforts providing services for creating and assigning identifiers? It would be helpful to clarify this by stating the specific efforts with which collaboration will take place.

We have revised the text to mention our work to align iSamples development with other international efforts.

In the last sentence of the text headed ‘iSamples solution’ the authors say with reference to figure 1 that to achieve the goals iSamples must advance standards and vocabularies across natural history domains. Was it intended to pick this domain specifically (which is generally taken to mean samples of plants and animals collected from the wild) or is the wider natural sciences domain what was really meant (i.e., samples of all biological (plants, animals, etc.) and non-biological (fossils, rocks, soil, etc.) materials occurring in the natural world)? I find this confusing at this point in reading the article although perhaps it becomes clearer later.

We have changed natural history to the natural sciences

In figure 1 the distinction between the blue (purple?) and purple (lilac?) dots is hard to make.

We have modified the colors. We also use an app that previews how colors look to people with different forms of color blindness, and modified the colors for maximum visibility across different vision profiles.

With a 10-reference limit on commentary articles it is not possible to reference everything mentioned. Nevertheless, it might be worthwhile to consider replacing the One Health reference [10] with one to CARE instead e.g., http://doi.org/10.5334/dsj-2020-043 as this is a critical new social consideration of making sample based data more accessible. Interconnecting disciplines is already well-known for some years.

In the technical description paragraph, the first sentence describing what iSamples Central is could be broken into two sentences after ‘discovery and retrieval’.

In the explanation of figure 2, the short-form words ‘ID’ and ‘sync’ are used when it would read better to spell out their long forms – identifier, synchronised.

Change comma to full-stop immediately after the reference to figure 3 and begin a new sentence.

Paragraph describing provenance. The last sentence would read better if it were broken after the phrase ‘…cannot always be inferred’ and the paratheses around the example forming the latter part of the present sentence were removed.

We have made these changes

Powered by Editorial Manager® and ProduXion Manager® from Aries Systems Corporation

	The screenshots forming parts of figure 3 would benefit from being taken from a larger screen so that the resolution can be improved at this PDF page scale.<br><br>We have improved the resolution<br><br>In the list of abbreviations, DataOne appears but it is not used in the main text.<br><br>The selected references are appropriate. However, most of them are missing the year of publication and a DOI.<br><br>We have removed DataOne from the abbreviations and updated references where possible<br><br>Reviewer #2:<br><br>...there have been recent efforts in the US and Europe to develop the concept of the Extended Specimen (in the US), and the Digital Specimen (in Europe). These relate to the standards, metadata schema and persistent identifiers for Natural History specimens, so only part of the domain purported to be covered by iSample, but given that iSample is focusing on this sector first, I would have expected some mention of this initiative, such as the recent GBIF consultation on this topic.<br><br>We added these concepts as an example of how iSamples will connect to broader efforts and to domains beyond natural science (we cite public health as one example). Recognizing this we changed the subtitle of the section from 'Beyond Natural History' to 'Beyond iSamples'.<br><br>the text makes a few assumptions that are probably beyond most readers working outside the domain. For example, the Traditional Knowledge and Biocultural Labels initiative is part of a "Local Contexts" project, but there is no citation, or explanation of why this is important.<br><br>We agree that there would ideally be a citation here but given the tightly limited number of references allowed, we believe the initiatives/projects mentioned can be easily found through an internet search. We have revised the text to provide a fuller (if still necessarily very brief) explanation of why it is important.<br><br>There are a few minor corrections needed in the text. Specifically:<br><br>- "significant sociological challenges remain to unleashing [should be unleash] the full value".<br>- "Integration of CARE as well as FAIR principles" [CARE is not defined in the text, just the abbreviation].<br><br>We have made these corrections<br><br>- "iSamples will focus on the natural history sector - any sample where geolocation is of primary importance" [I don't understand the context of the geolocation reference or its specific link to natural history collections - its relevant to all collections at some level].<br><br>We have removed "any sample where geolocation is of primary importance"
**Additional Information:**	
**Question**	**Response**
Are you submitting this manuscript to a special series or article collection?	No
**Experimental design and statistics**<br><br>Full details of the experimental design and statistical methods used should be given	Yes

Powered by Editorial Manager® and ProduXion Manager® from Aries Systems Corporation

in the Methods section, as detailed in our Minimum Standards Reporting Checklist. Information essential to interpreting the data presented should be made available in the figure legends.<br><br>Have you included all the information requested in your manuscript?	
**Resources**<br><br>A description of all resources used, including antibodies, cell lines, animals and software tools, with enough information to allow them to be uniquely identified, should be included in the Methods section. Authors are strongly encouraged to cite Research Resource Identifiers (RRIDs) for antibodies, model organisms and tools, where possible.<br><br>Have you included the information requested as detailed in our Minimum Standards Reporting Checklist?	Yes
**Availability of data and materials**<br><br>All datasets and code on which the conclusions of the paper rely must be either included in your submission or deposited in publicly available repositories (where available and ethically appropriate), referencing such data using a unique identifier in the references and in the "Availability of Data and Materials" section of your manuscript.<br><br>Have you have met the above requirement as detailed in our Minimum Standards Reporting Checklist?	Yes

Manuscript
Click here to access/download;Manuscript;Revised_iSamples Marker paper.docx

Internet of Samples (iSamples): Toward an Interdisciplinary Cyberinfrastructure for Material Samples

- Neil Davies[1,2]; ndavies@berkeley.edu; https://orcid.org/0000-0001-8085-5014
- John Deck[3]; jdeck@berkeley.edu; https://orcid.org/0000-0002-5905-1617
- Eric C. Kansa[4]; kansaeric@gmail.com; https://orcid.org/0000-0001-5620-4764
- Sarah Whitcher Kansa[4]; skansa@alexandriaarchive.org; https://orcid.org/0000-0001-7920-5321
- John Kunze[5], jak@ucop.edu; https://orcid.org/0000-0001-7604-8041
- Christopher Meyer[6], meyerc@si.edu; https://orcid.org/0000-0003-2501-7952
- Thomas Orrell[6]; orrellt@si.edu; https://orcid.org/0000-0003-1038-3028
- Sarah Ramdeen[7], sramdeen@ldeo.columbia.edu; https://orcid.org/0000-0003-1135-5942
- Rebecca Snyder[6] , snyderr@si.edu, https://orcid.org/0000-0002-0028-6139
- Dave Vieglais[8]; vieglais@ku.edu; https://orcid.org/0000-0002-6513-4996
- Ramona L. Walls[9], rwalls@email.arizona.edu; https://orcid.org/0000-0001-8815-0078
- Kerstin Lehnert[7]; lehnert@ldeo.columbia.edu; https://orcid.org/0000-0001-7036-1977

1. Gump South Pacific Research Station, University of California, BP 244 98728, Moorea, French Polynesia
2. Berkeley Institute for Data Science, University of California, Berkeley, CA 94720, USA
3. Berkeley Natural History Museums, University of California, Berkeley, CA 94720, USA
4. Open Context, The Alexandria Archive Institute, San Francisco, CA, 94127, USA
5. California Digital Library, University of California, Office of the President, Oakland, CA 94607, USA
6. National Museum of Natural History, Smithsonian Institution, Washington DC, 20560, USA
7. Lamont-Doherty Earth Observatory, Columbia University, Palisades, NY 10964, USA
8. Biodiversity Institute, The University of Kansas, KS, 66045, USA
9. Bio5 Institute, University of Arizona, Tucson, AZ 85718, USA

Abstract

Sampling the natural world and built environment underpins much of science, yet systems for managing material samples and associated (meta)data are fragmented across institutional catalogs, practices for identification, and discipline-specific (meta)data standards. The Internet of Samples (iSamples) is a standards-based collaboration to uniquely, consistently, and conveniently identify material samples, record core metadata about them, and link them to other samples, data, and research products. iSamples extends existing resources and best practices in data stewardship to render a cross-domain cyberinfrastructure that enables transdisciplinary research, discovery, and reuse of material samples in 21st century natural science.

Keywords

Material sample, specimen, data standards, cyberinfrastructure, unique identifiers, persistent identifiers, collections, geoscience, bioscience, archaeology

Background

Material samples from natural and built environments are fundamental to many branches of science and are increasingly needed for interdisciplinary research with critical societal relevance, such as sustaining natural resources, controlling infectious diseases, and coping with environmental change. Scientific collections have entered the realm of big data with the advent of simultaneous sampling across large areas and repeated sampling of the same area [1–3]. Many (perhaps most) material samples, however, are not accessioned into institutional collections but remain ‘hidden’ in labs, offices, and basements, as researchers and institutions often lack the resources and expertise to properly curate them [4]. Harnessing existing sample-based data for science is cumbersome and often impractical as data about most material samples are difficult or impossible to Find, Access, Interoperate, and Reuse -- they are simply not FAIR [5]. As a consequence, the full value of material samples and the data derived from them is rarely realized, either for basic scientific research or societal applications. For example, published DNA sequence data often lack the geographic metadata needed to understand the origin and spread of pathogens [6]. Maximizing the value of today’s samples for tomorrow’s science requires cyberinfrastructure designed to facilitate sharing and reuse across the material sample value chain and to accommodate the interdisciplinary nature of many samples (**Box 1**). Unleashing societal benefits from material samples requires linking them to derived data and published interpretations of those data; also essential steps to making sample-based scientific knowledge reproducible, credible, and useful. In order to achieve these linkages, material samples need globally unique, persistent, and resolvable identifiers with reliably accessible and trustable standards-based metadata describing the sample and its provenance. Finally, sample cyberinfrastructure must ease frictions of software (machines) interacting with the (meta)data.

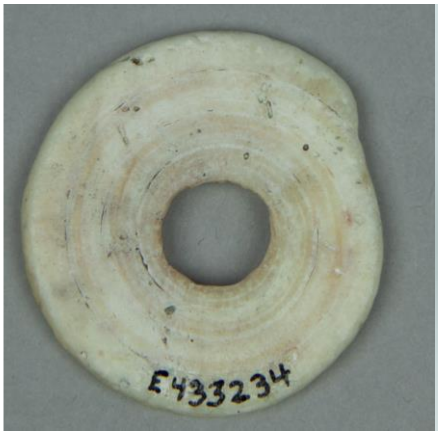

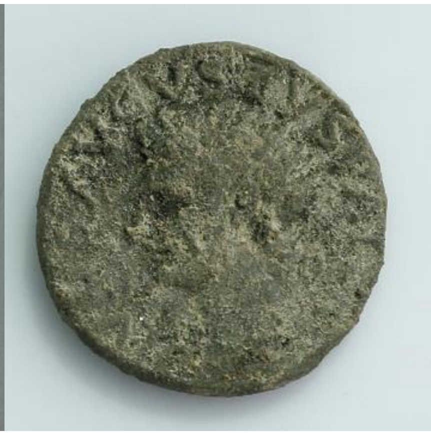

Box 1: Interdisciplinarity of Material Samples - Example from Archaeology

Archaeologists study highly diverse material culture created over many millennia by peoples across the world who lived in very different regions, societies, and cultural traditions. While material culture is difficult to describe with standard metadata, archaeologists draw upon geological and biological sources of evidence and vice versa. For example, large-scale data integration of animal remains has been used to demonstrate domestication patterns in Southwest Asia [7]. It is vital, however, that samples have appropriate provenance information and other metadata. Take the case of a research program investigating the ancient use of coins. Coins are "samples", which should have persistent identifiers and metadata about time, space, and other aspects of archaeological context. Samples of 'biological' coins, such as shell money (see photo on left; E433234, Department of Anthropology, Smithsonian Institution), might also yield useful information for biologists, such as the historical biogeography of species. Similarly, samples of metal coins (see photo on right; Opitz, Mogetta, and Terrenato. Sp.Find 956, The Gabii Project: Open Context. ARK:https://n2t.net/ark:/28722/k2697cp2q) have important geological aspects. Numismatists use mint-marks and iconography to infer location and date of a coin's manufacture, while geoscientists can characterize the same coin with isotope studies to allow investigation of the ore sources and post-depositional processes. iSamples will provide the cyberinfrastructure needed to facilitate such connections within and across scientific domains.

Main text

iSamples Solution

Recognizing the need for research infrastructure to support material samples, the U.S. National Science Foundation funded iSamples in 2020 to develop consistent services for unique and persistent sample identification and sample metadata registration across disciplines. Complementing related efforts globally, such as those of Australia's national science agency (CSIRO) and Europe's Distributed System of Scientific Collections (DiSSCo), iSamples will provide services for creating and assigning persistent, unique, and resolvable identifiers to material samples in a consistent manner across disciplines, and for registering and indexing metadata using semantic web technologies. The result will be a searchable global index of material samples linked to appropriate metadata and derived data products. iSamples aims to (i) enable previously impossible connections between diverse and disparate sample-based observations; (ii) support existing research programs and facilities that collect and manage diverse sample types; (iii) facilitate new interdisciplinary collaborations; and (iv) provide an efficient solution for FAIR samples, avoiding duplicate efforts in different domains. To achieve its goals, iSamples must incorporate and help advance diverse metadata vocabularies and standards across natural science domains (**Figure 1**).

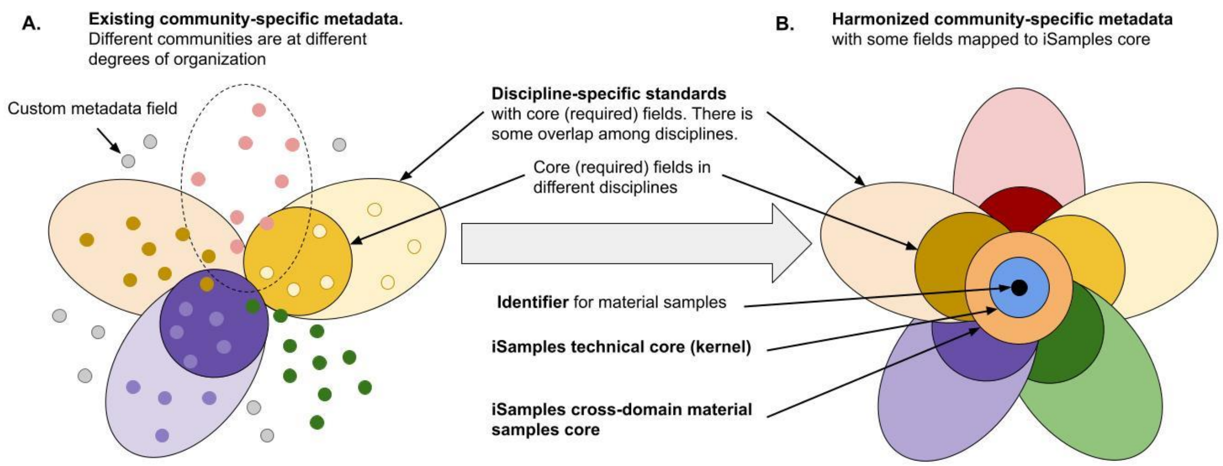

Figure 1. iSamples Vision: Metadata in Bloom

iSamples will extend existing domain- and sample-specific efforts, rendering a cross-domain cyberinfrastructure that can serve all samples from the natural and built environment. **(A)** Currently, each discipline creates its own community-specific metadata fields (different colored dots) and data standards (oval shaped 'petals') based on their specialized knowledge and needs. These apply to material samples and/or a range of digital objects such as photos, datasets, genetic sequences, and publications. Disciplinary communities are at different stages of organization. The most advanced have standardized metadata fields (e.g., yellow, purple, and tan petals) sometimes with minimum required fields (darker inner petals - known as cores or kernels). Some disciplines are beginning to organize (pink dots with dotted-line petal) while others have no organization as yet (green dots). Some metadata fields cut across disciplines (the brown and green dots in the purple domain). At the cutting edge of research, new data types and custom metadata fields are constantly emerging (gray dots). **(B)** Cyberinfrastructure being built by iSamples focuses on sampling events and the resulting material samples and subsamples thereof. Metadata needed will include the material sample identifier (black dot) and its required technical core or kernel (light blue circle), as well as an iSamples cross-domain core (orange circle) that encompasses all required metadata fields shared across disciplines in the natural sciences. Promoting and facilitating community-driven metadata standards from each domain, iSamples will also support the creation of interdisciplinary metadata profiles (see Figure 2, iSamples-in-a-Box) that include metadata fields from the iSamples core to serve the needs of interdisciplinary researchers and other users.

Technical Description: Distributed Cyberinfrastructure

The iSamples system has two core components (**Figure 2**). An **iSamples-in-a-Box** instance is a standalone system that enables creation of identifiers and associated metadata, retrieval of the sample information, updates to the sample metadata (e.g., augmenting or correcting metadata or appending provenance statements), sample identifier resolution, and discovery of samples. iSamples-in-a-Box will support different scenarios. Initial use cases include: (a) SESAR, which provides reliable services for sample metadata cataloguing and Global Sample Number (IGSN) registration for individual researchers and institutions [8]; (b) GEOME, which supports capturing metadata on biological samples and links to associated genomic data [9]; and (c) Open Context, a publishing service maintained by the Alexandria Archive Institute, which serves as a metadata repository for archaeological artefacts and ecofacts and links samples to associated data. **iSamples Central** is designed as a permanent Internet service that preserves and indexes sample metadata to ensure reliable discovery and retrieval. It provides a gateway between iSamples-in-a-Box instances and identifier authorities to ensure that remote iSamples-in-a-Box content is fully synchronized with the relevant authorities (e.g., IGSNs generated on iSamples-in-a-Box are synchronized with iSamples Central and the IGSN central authority). By offering services that augment existing identifier authority capabilities, iSamples Central enables support of other identifier types such as ARKs or DOIs that are not traditionally associated with material samples, but are used by some organizations. iSamples Central is a central discovery and resolution service (search interface on the web and API) for any community that wishes to participate, while iSamples-in-a-Box will deliver distributed infrastructure early in the data production chain with an emphasis on the needs of specific research domains.

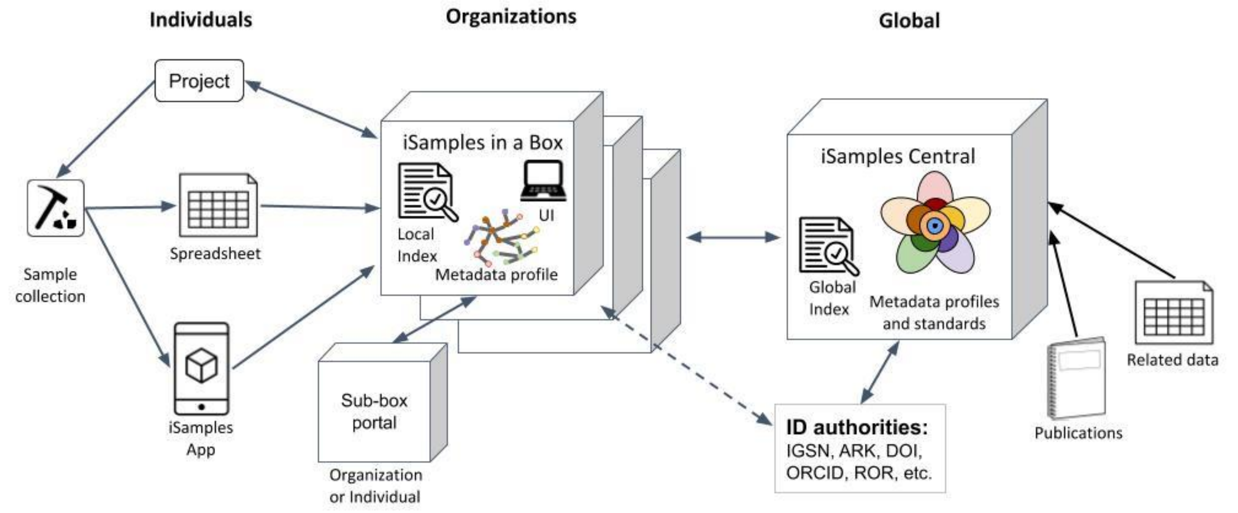

Figure 2. iSamples System Infrastructure

iSamples infrastructure supports individuals and organizations through two key components. The iSamples project will create generic code that can be used to build many instances of **iSamples-in-a-Box** (center). Each box is a domain or community portal that provides local services for identifier allocation and metadata collection according to metadata profiles specific to that portal. iSamples-in-a-Box would either use the existing qualifying identifiers, or in the case of non-qualifying legacy (non-unique) identifiers, generate new identifiers as needed and link them (sameAS). Individual users will push their sample metadata, collected via spreadsheets or apps (left), to the iSamples-in-a-box local index. Larger institutions may choose to create sub-boxes (e.g., a museum might create a sub-box for its field station). Boxes connect to **iSamples Central** (right) to verify their accounts with identifier authorities, download or synchronize metadata profiles, and -- if they choose -- to synchronize their metadata with the iSamples Central global index for discovery, resolution, and identifier coordination (ensuring that newly minted identifiers are associated with minimal metadata and that such records are collated locally and globally). iSamples Central manages cross-disciplinary metadata according to the model described in Figure 1B. The iSamples Central index also stores links to related data and publications: Records collated within the iSamples infrastructure are parsed to extract related objects and their predicates to determine explicit internal relations. Explicit external relations (i.e., references to entities outside of iSamples) are also collated though may be more fragile. Implicit relations are inferred by similarity of record attributes (e.g., records within a spatio-temporal region have an inferred relationship). Relations to publications requires that identifiers contained within publications are readily available, and this requires coordination with publishers to ensure extraction of the necessary information (minimally a list of identifiers occurring within a publication). Emerging infrastructure, such as the EventData service provided by Crossref, are starting to provide such capabilities on a large scale.

Provenance is often truncated in current data systems (**Figure 3**). iSamples takes an event-based approach capturing metadata upstream from Field Information Management Systems and maintaining links downstream, with metadata standards implemented or inferred at each step. Some metadata are inferred, as they must follow all parent-child relationships (e.g., 'where' and 'when' of the collecting event), but other types of metadata (e.g., taxonomy) cannot always be inferred. For example, a subsample from a fish might not inherit the fish's taxonomy as it might be something the fish ate or a parasite; similarly, a mineral subsampled from a rock cannot inherit the rock taxonomy.

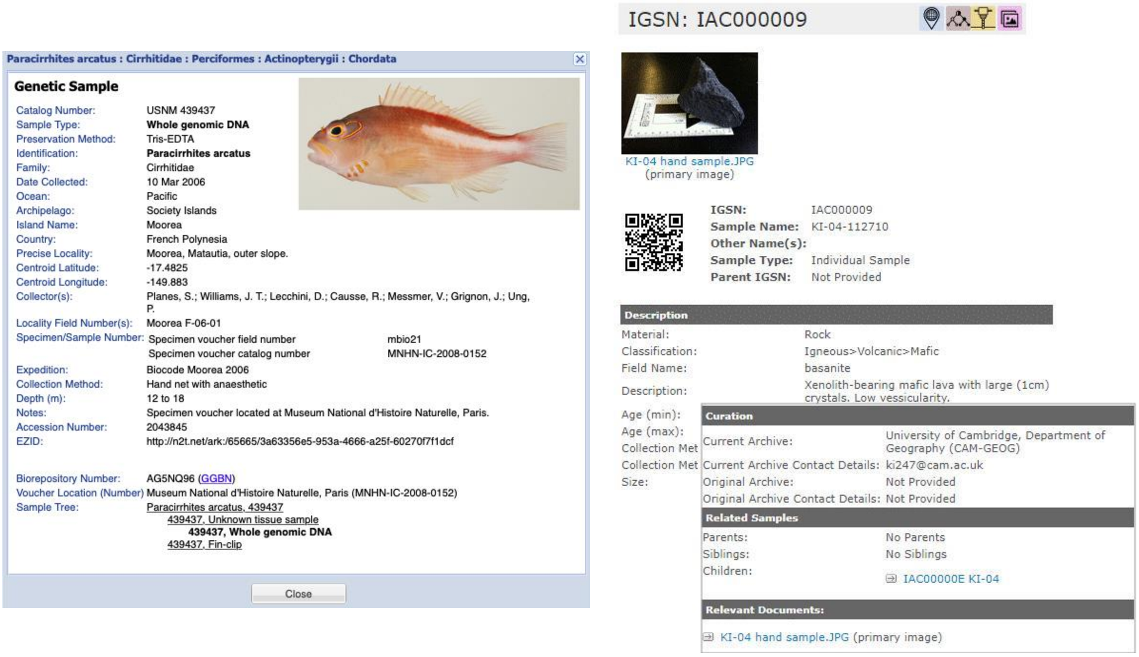

Figure 3. Provenance tracking - the iSample Tree.

The record on the left is for a Genetic Sample in the Smithsonian's NMNH Biorepository (AG5NQ96). Each material sample has its own identifier, in this case an EZID ARK (http://n2t.net/ark:/65665/3a63356e5-953a-4666-a25f-60270f7f1dcf). The DNA was extracted from a tissue (439437, Unknown tissue sample) that was taken from a fish (Paracirrhites arcatus, 439437) that can be found (voucher specimen) in the National Museum of Natural History in Paris as catalog number MNHN-IC-2008-0152. The "Sample Tree" field reveals the provenance of the DNA, and also reveals another tissue sample (439437, Fin-clip) that was taken from the same fish. Clicking on any sample in the sample tree would reveal its own EZID ARK.

The record on the right is for a rock sample, KI-04-112710, registered in the SESAR catalog by a research scientist. Each sample registered in SESAR is assigned an IGSN as a unique identifier, in this case IAC000009. KI-04-112710 is a hand sample that is representative of primitive lavas from Antarctica and was subsequently powdered for additional analysis. In the "Related Samples" field of its profile page, the resulting rock powder is listed as a child sample, KI-04-11272010 (IAC00000E). KI-04-11272010 was resampled for phase equilibrium experiments and lists 27 children samples. The links between the records provides the provenance between the parent, child, and grandchildren samples. By registering identifiers, iSamples will enable such "Sample Trees" to reveal a larger value chain, such as linking collecting events to other specimens and their derivatives and resulting data.

Sampling Nature: Sustainability, Inclusion, and Equity

While iSamples has funding to build cyber-infrastructure addressing technological barriers, significant sociological challenges must be overcome to unleash the full value of material samples. iSamples will engage scientific and technical expertise around standards and ontologies (e.g., through Research Coordination Network mechanisms). Harnessing material samples for sustainable development, however, also requires empowering a broad swath of stakeholders to benefit from material samples, related data and research products - particularly people from whose communities the samples are derived. It is vital that standards, training materials, public outreach, and policy recommendations are equitable and inclusive. Important areas of emphasis include Access and Benefit Sharing (Convention on Biological Diversity), Indigenous data rights and social justice, where inequities of the past and present need to be addressed. Key steps that iSamples will pursue include the integration of Collective Benefit, Authority to Control, Responsibility, and Ethics - the ‘CARE principles’ [10] and the adoption of Traditional Knowledge & Biocultural Labels and Notices, an initiative of “Local Contexts” that provides a mechanism for Indigenous communities to engage with cultural and research institutions to manage their traditional rights over their property and knowledge.

Beyond iSamples

The need for permanent identifiers and robust metadata is not unique to material samples. Building a fully-comprehensive internet of samples will require infrastructure similar to iSamples for all resources connected to samples, including datasets, images, sound recordings, and publications. iSamples will contribute to such efforts, for example, around the concepts of Digital Specimens and networks of Extended Specimens. Furthermore, while iSamples focuses on the natural sciences, material samples are important in several sectors that are increasingly interconnected, such as approaches to public health that combine ecology and medicine.

Conclusions

iSamples will allow scientists to track natural science samples, subsamples, associated metadata, data, and research products. iSamples is a single, distributed, transdisciplinary infrastructure based on domain-neutral technologies, standards, and consistent sample identification that is extensible to accommodate domain-specific needs. iSamples aims to enhance existing research within disciplines while enabling new research across them.

List of abbreviations

ARK: Archival Resource Key
CARE: Collective benefit, Authority to control, Responsibility, Ethics
DOI: Digital Object Identifier
FAIR: Findable, Accessible, Interoperable, Reusable
GEOME: Genomic Observatories Metadatabase
GSC: Genomic Standards Consortium
IGSN: IGSN Global Sample Number

ORCID: Open Researcher and Contributor ID
ROR: Research Organization Registry
SESAR: System for Earth Sample Registration

Declarations

Data availability

Not applicable.

Consent for publication

Not applicable.

Competing interests

The authors declare that they have no competing interests.

Funding

This material is based upon work supported by the National Science Foundation under Grant Numbers 2004839, 2004562, 2004642, and 2004815. Any opinions, findings, and conclusions or recommendations expressed in this material are those of the author(s) and do not necessarily reflect the views of the National Science Foundation.

Authors' contributions

Much of the text is derived from the collaborative "iSamples" proposal that was submitted to the National Science Foundation following a workshop KL organized in August 2019 at Columbia University. ND put together the first draft of the current manuscript and all authors contributed to subsequent drafts, with RW adding Figure 1, DV and RW Figure 2, and CM Figure 3. All authors read and approved the final manuscript.

Acknowledgements

We are grateful for input on the draft manuscript from Hong Cui and Stephen Richard, and to Leslie Weyborn for inspiration for our metadata model and Figure 1.

References

1. Davies N, Field D, Amaral-Zettler L, Clark MS, Deck J, Drummond A, et al.. The founding charter of the Genomic Observatories Network. *Gigascience*. 2014; doi: 10.1186/2047-217X-3-2.

2. Buttigieg PL, Janssen F, Macklin J, Pitz K. The Global Omics Observatory Network: Shaping standards for long-term molecular observation. *Biodiversity Information Science and Standards*. Pensoft Publishers; 2019; doi: 10.3897/biss.3.36712.

3. Mirtl M, T Borer E, Djukic I, Forsius M, Haubold H, Hugo W, et al.. Genesis, goals and achievements of Long-Term Ecological Research at the global scale: A critical review of ILTER and future directions. *Sci Total Environ*. 2018; doi: 10.1016/j.scitotenv.2017.12.001.

4. McNutt M, Lehnert K, Hanson B, Nosek BA, Ellison AM, King JL. Liberating field science samples and data. *Science*. American Association for the Advancement of Science; 2016; doi: 10.1126/science.aad7048.

5. Wilkinson MD, Dumontier M, Aalbersberg IJJ, Appleton G, Axton M, Baak A, et al.. The FAIR Guiding Principles for scientific data management and stewardship. *Sci Data*. 2016; doi: 10.1038/sdata.2016.18.

6. Schriml LM, Chuvochina M, Davies N, Eloe-Fadrosh EA, Finn RD, Hugenholtz P, et al.. COVID-19 pandemic reveals the peril of ignoring metadata standards. *Scientific Data*. 2020; doi: 10.1038/s41597-020-0524-5.

7. Arbuckle BS, Kansa SW, Kansa E, Orton D, Çakırlar C, Gourichon L, et al.. Data sharing reveals complexity in the westward spread of domestic animals across Neolithic Turkey. *PLoS One*. 2014; doi: 10.1371/journal.pone.0099845.

8. Ramdeen S, Lehnert K, Markey K, Devendran S, Johansson A, Song L. Citations for physical samples: IGSN and the System for Earth Sample Registration. p. IN12B – 01.

9. Deck J, Gaither MR, Ewing R, Bird CE, Davies N, Meyer C, et al.. The Genomic Observatories Metadatabase (GeOMe): A new repository for field and sampling event metadata associated with genetic samples. *PLoS Biol*. 2017; doi: 10.1371/journal.pbio.2002925.

10. Carroll SR, Garba I, Figueroa-Rodríguez OL, Holbrook J, Lovett R, Materechera S, et al.. The CARE principles for indigenous data governance. *Data Sci J*. Ubiquity Press, Ltd.; 2020; doi: 10.5334/dsj-2020-043.

Figure 1 Click here to access/download;Figure;Fig-1_iSamples metadata.jpeg

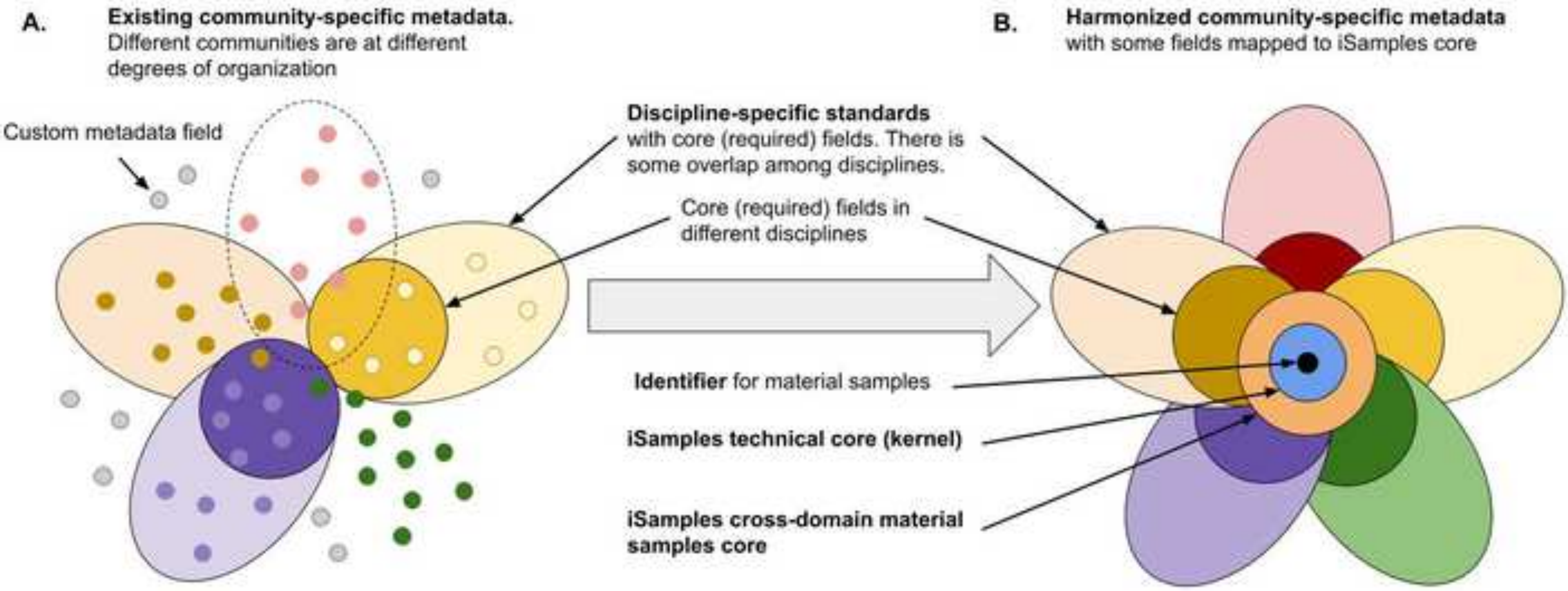

Figure 3

Click here to access/download;Figure;Fig3_iSamples marker.tiff

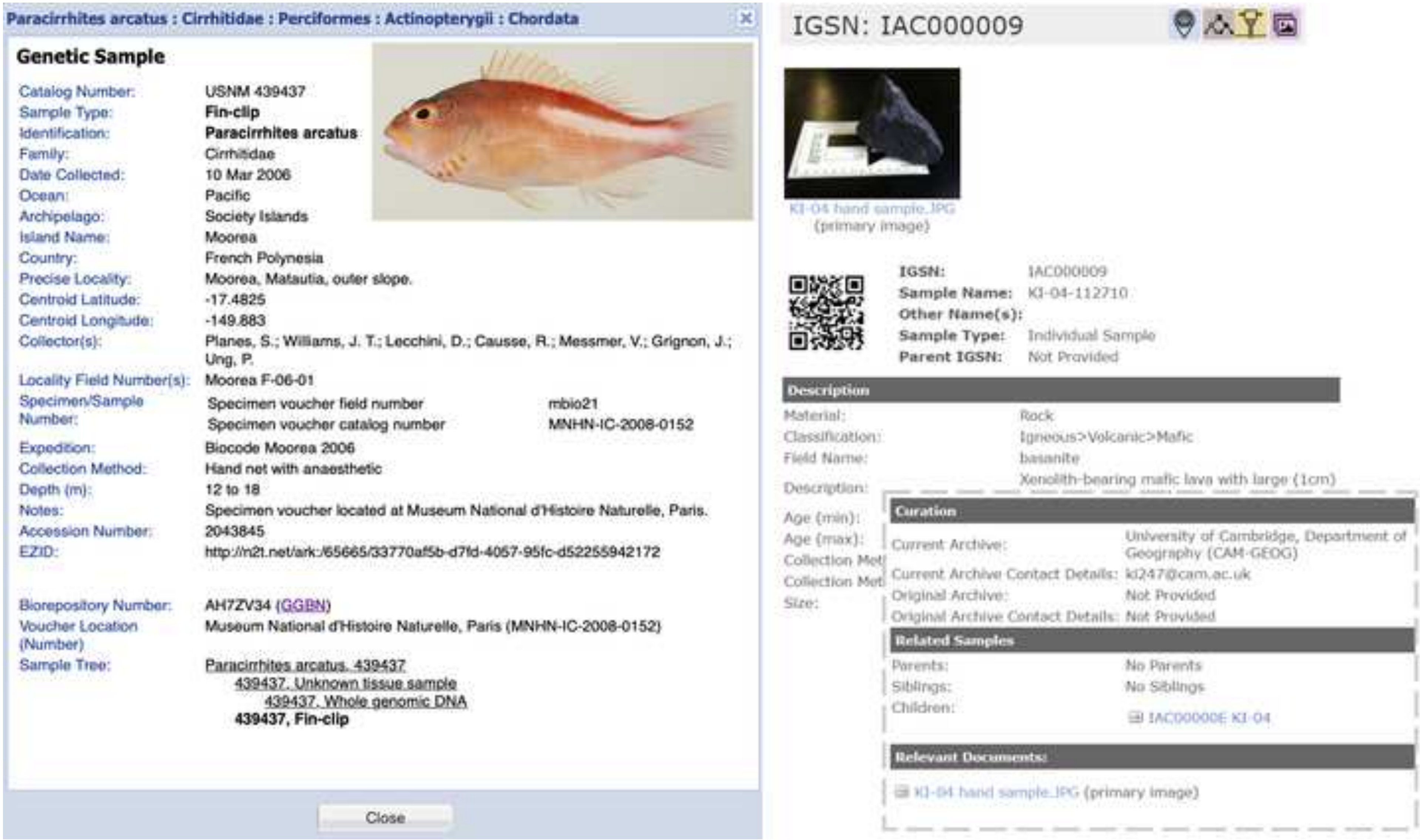

Personal Cover
Click here to access/download;Personal Cover;Response to Reviewers_iSamples.docx

Internet of Samples (iSamples): Toward an Interdisciplinary Cyberinfrastructure for Material Samples

Response to Reviewers

We are grateful to both reviewers for their careful consideration of this manuscript and below we provide point-by-point responses (bullet points) to their comments (in *italics*):

Reviewer #1:

Figure 3. … the explanation of the provenance (sample tree) relations among the things is correct but the assertions that the numbers in the sample tree are ARKs is incorrect. This explanation needs to be revised.

- Figure 3: we have corrected the problems identified with the numbers in the sample tree

In figure 1 a distinction is made between iSamples digital objects and iSamples physical objects but no explanation is given of the difference. What is meant by the former?

- We intend to eventually extend iSamples to digital objects, and the ID will be required for these as well. However, this paper simply describes our work for material samples, so we have simplified the language in figure 1 and its legend.

Also in figure 1, it is mentioned that facilitating community- driven metadata standards and adopting interdisciplinary metadata profiles will take place. These are social challenges. Later in the article, there is an admission that significant social challenges exist but not much is said about how the project aims to tackle these.

- We intend to address these challenges through a Research Coordination Network "Sampling Nature". We have not cited this RCN directly, however, because the award is not yet finalized. Instead, we have revised the text to mention the importance of RCNs as a mechanism for engaging the community in the social aspects of cyberinfrastructure.

In the explanation of figure 2 the phrase 'identifier coordination' is used. What is meant by this?

- Identifier coordination involves ensuring that newly minted identifiers are associated with minimal metadata and that such records are collated locally and globally. We have added this additional explanation to the Figure 2 caption.

It is said that iSamples Central index also stores links to related data and publications as well as the metadata about the samples. How these links will be captured and created is not explained.

- We have added an explanation to the Figure 2 legend.

No mention is made… organization- specific (legacy) identifier types arising from historic collections ...How can these cases of non- unique identifiers be accommodated by iSamples?

- We have updated the text in the Figure 2 legend to address this issue (briefly). A fuller response: While legacy non-unique identifier types are an important issue, iSamples is focused on the need for all new samples to follow best-practice in assigning unique identifiers and thus not to contribute to the scale of the problem going forward. Having said that, assurance of legacy record uniqueness can be achieved through namespacing (e.g., prefixing with an ARK shoulder) or minting of a new identifier. We plan to take advantage of the California Digital Library N2T (name to thing) resolver service to help manage legacy identifiers with an option to convert legacy identifiers into globally unique “compact identifiers”. [https://doi.org/10.1038/sdata.2018.95] In either case, the original identifier is retained with a "sameAs" relation to ensure ongoing resolution, albeit with potentially multiple matches that require additional refinement. iSamples in-a-box services would allow legacy institutions without identifier best practices to participate.

...how will machine/software- oriented rather than human- oriented processing of digital data about samples be catered for. The article does not discuss machine- actionability of sample data at all; although the authors acknowledge that further work beyond the present project will be required.

- We very much agree that machine-actionability is a key issue and iSamples represents a first step by making sample data FAIRer, and as the reviewer points out, the FAIR principles are themselves aimed at machines. We added a sentence recognizing that sample cyberinfrastructure must strive to ease frictions of software (machines) interacting with the (meta)data.

Access and benefit sharing should be mentioned alongside CARE.

- We have made this addition in the text

In the text headed ‘iSamples solution’: The authors say iSamples will be ‘collaborating with similar efforts globally’ but they do not state/reference what these efforts are. The reader cannot infer what is meant here. Are similar efforts for identifying and indexing samples meant or is it similar efforts providing services for creating and assigning identifiers? It

would be helpful to clarify this by stating the specific efforts with which collaboration will take place.

- We have revised the text to mention our work to align iSamples development with other international efforts.

In the last sentence of the text headed 'iSamples solution' the authors say with reference to figure 1 that to achieve the goals iSamples must advance standards and vocabularies across natural history domains. Was it intended to pick this domain specifically (which is generally taken to mean samples of plants and animals collected from the wild) or is the wider natural sciences domain what was really meant (i.e., samples of all biological (plants, animals, etc.) and non- biological (fossils, rocks, soil, etc.) materials occurring in the natural world)? I find this confusing at this point in reading the article although perhaps it becomes clearer later.

- We have changed natural history to the natural sciences

In figure 1 the distinction between the blue (purple?) and purple (lilac?) dots is hard to make.

- We have modified the colors. We also use an app that previews how colors look to people with different forms of color blindness, and modified the colors for maximum visibility across different vision profiles.

With a 10- reference limit on commentary articles it is not possible to reference everything mentioned. Nevertheless, it might be worthwhile to consider replacing the One Health reference [10] with one to CARE instead e.g., http://doi.org/10.5334/dsj- 2020- 043 as this is a critical new social consideration of making sample based data more accessible. Interconnecting disciplines is already well- known for some years.

In the technical description paragraph, the first sentence describing what iSamples Central is could be broken into two sentences after 'discovery and retrieval'.

In the explanation of figure 2, the short- form words 'ID' and 'sync' are used when it would read better to spell out their long forms – identifier, synchronised.

Change comma to full- stop immediately after the reference to figure 3 and begin a new sentence.

Paragraph describing provenance. The last sentence would read better if it were broken after the phrase '…cannot always be inferred' and the paratheses around the example forming the latter part of the present sentence were removed.

- We have made these changes

The screenshots forming parts of figure 3 would benefit from being taken from a larger screen so that the resolution can be improved at this PDF page scale.

- We have improved the resolution

In the list of abbreviations, DataOne appears but it is not used in the main text.

The selected references are appropriate. However, most of them are missing the year of publication and a DOI.

- We have removed DataOne from the abbreviations and updated references where possible

Reviewer #2:

...*there have been recent efforts in the US and Europe to develop the concept of the Extended Specimen (in the US), and the Digital Specimen (in Europe). These relate to the standards, metadata schema and persistent identifiers for Natural History specimens, so only part of the domain purported to be covered by iSample, but given that iSample is focusing on this sector first, I would have expected some mention of this initiative, such as the recent GBIF consultation on this topic*.

- We added these concepts as an example of how iSamples will connect to broader efforts and to domains beyond natural science (we cite public health as one example). Recognizing this we changed the subtitle of the section from ‘Beyond Natural History’ to ‘Beyond iSamples’.

the text makes a few assumptions that are probably beyond most readers working outside the domain. For example, the Traditional Knowledge and Biocultural Labels initiative is part of a "Local Contexts" project, but there is no citation, or explanation of why this is important.

- We agree that there would ideally be a citation here but given the tightly limited number of references allowed, we believe the initiatives/projects mentioned can be easily found through an internet search. We have revised the text to provide a fuller (if still necessarily very brief) explanation of why it is important.

There are a few minor corrections needed in the text. Specifically:

- "significant sociological challenges remain to unleashing [should be unleash] the full value".

- "Integration of CARE as well as FAIR principles" [CARE is not defined in the text, just the abbreviation].

- We have made these corrections

- "iSamples will focus on the natural history sector - any sample where geolocation is of primary importance" [I don't understand the context of the geolocation reference or its specific link to natural history collections - its relevant to all collections at some level].

- We have removed “any sample where geolocation is of primary importance”
