# Supplementary material for: Internet of Samples (iSamples): Toward an interdisciplinary cyberinfrastructure for material samples
Source: Gigascience. 2021 May 7;10(5):giab028. doi: 10.1093/gigascience/giab028 (PMC8103498; doi:10.1093/gigascience/giab028)
Supplement: giab028_Alex_Hardisty_Review_iSamples_04Mar2021 — Alex Hardisty -- 3/4/2021 Reviewed [file giab028_alex_hardisty_review_isamples_04mar2021.pdf]

Reviewer's Overall Assessment

Date 04 March 2021

Manuscript # GIGA-D-21-00056

Title Internet of Samples (iSamples): Toward an Interdisciplinary Cyberinfrastructure for Material Samples

Manuscript Type Commentary

Revisions required Minor

Rationale

The commentary describes a vision for collaboratively working towards a standards-based cross-domain cyberinfrastructure for the identification of material samples of multiple kinds, the consistent recording of metadata about those samples and the linking of them to data artefacts.

The authors explain that material samples of all kinds are becoming more important to modern science, for solving challenges such as sustaining natural resources, coping with environmental change, and controlling infectious diseases. They explain the difficulties related to accessing samples and the importance of making samples more readily accessible and more reusable by properly identifying them, describing them and indexing them. Maximising and unleashing the societal value of material samples and allowing them to become better linked to derived data and interpretations can be achieved through cyber/e-infrastructure designed to facilitate sharing and reuse. The authors propose that material samples need globally unique, persistent and resolvable identifiers with reliable, accessible and trustable standards-based metadata. This rationale is clearly explained as the background and examples are given from archaeology (coins), natural history (fish DNA) and geology (powdered rock). The authors explain that iSamples will set out to build a distributed infrastructure consisting of locally deployable capability (iSamples-in-a-Box) for creating identifiers and capturing metadata and 'iSamples Central' – a searchable global index of material samples linked to appropriate metadata and derived data products that is created by harvesting from multiple iSamples-in-a-Box instances.

Are the data sound/well controlled?

As a commentary article there is little data referenced or provided. However, there are some illustrative examples with typical data given in figures with some explanations. In one case (figure 3, fish DNA example) the explanation is not sound.

In figure 3 it is explained that the genetic sample in the Smithsonian's Biorepository has its own identifier – in the example, an EZID ARK that's bolded in the relational tree i.e., 439437. After having ascertained the Smithsonian's ARK NAAN as being 65665 I attempted to resolve this ARK as follows: http://n2t.net/ark:/65665/439437. Redirection to the Search Museum Collection Records of the Smithsonian took place and an error message "The ARK you requested was not found" was returned. Going to the Division of Fishes search page (https://collections.nmnh.si.edu/search/fishes/) and entering '439437' as the Catalog Number returned four results, including the one used as the Figure 3 example (left). So, '439437' does not appear to be a valid ARK, and in fact this catalog number identifies four different things derived from the original MNHN specimen. Inspection of each of the pages for the four things reveals that each has its own EZID ARK of the form ark:/65665/<identifier-string>. These do resolve correctly. Thus, the explanation of the provenance (sample tree) relations among the things

is correct but the assertions that the numbers in the sample tree are ARKs is incorrect. This explanation needs to be revised.

Checking the IGSN example (figure 3, right side), the IGSN IAC000009 resolves correctly i.e., http://igsn.org/IAC000009 redirects to https://app.geosamples.org/sample/igsn/IAC000009 to display the profile page, part of which is shown in figure 3. The further explanation of identifiers and relations between samples is sound.

Is the interpretation (analysis and discussion) well balanced?

The explanations provided by the article are clear and reasonable. The authors have done a good job within the constraints of the guidelines for commentary articles. The reader will gain a good sense of the scope and intention of iSamples, as well as the benefits that may come from the initiative.

Are the methods appropriate, well described and with enough details?

In general, yes. Not everything can be discussed in an article of this length. There are some places where the reader might be left with questions, for example:

- In figure 1 a distinction is made between iSamples digital objects and iSamples physical objects but no explanation is given of the difference. What is meant by the former?
- Also in figure 1, it is mentioned that facilitating community-driven metadata standards and adopting interdisciplinary metadata profiles will take place. These are social challenges. Later in the article, there is an admission that significant social challenges exist but not much is said about how the project aims to tackle these.
- In the explanation of figure 2 the phrase 'identifier coordination' is used. What is meant by this?
- It is said that iSamples Central index also stores links to related data and publications as well as the metadata about the samples. How these links will be captured and created is not explained.

What are the strengths and weaknesses?

iSamples, with its two components of 'in-a-Box' and 'Central' offers a coherent approach to the problems of identifying, describing and indexing material samples in the future. IGSNs are the primary identifier type being supported and promoted. It is acknowledged that other kinds of modern identifiers in the digital realm, such as ARK and DOI can also be accommodated.

No mention is made, however of the huge numbers of organization-specific (legacy) identifier types arising from historic collections that have been using their own sample (specimen) identifiers for more than 200 years. Not being individually unique makes these a particular problem to deal with as there is no appetite to go back and re-identify everything. Identifying codes on physical specimens (e.g., handwritten catalog numbers, barcodes, etc.) must not be conflated with identifiers (i.e., URLs) of publicly available catalogue records about those specimens. They are not the same thing. How can these cases of non-unique identifiers be accommodated by iSamples?

As the numbers of samples, links between them and to/from derived data data products increases substantially in the future, how will machine/software-oriented rather than human-oriented processing of digital data about samples be catered for. The FAIR Guiding Principles are intended not only to ease the life of humans but also strongly emphasize making machine-processing 'FAIRer'. The article does not discuss machine-actionability of sample data at all; although the authors acknowledge that further work beyond the present project will be required.

Access and benefit sharing should be mentioned alongside CARE.

Have the authors followed best-practices in reporting standards?

Not applicable.

Can the writing, organization, tables and figures be improved?

The title and chosen keywords are appropriate. The abstract is succinct and clear. The article is of an appropriate length and is generally clear and easy to read. The following issues, when addressed would increase the overall readability and lead to easier understanding by the reader:

- In the text headed 'iSamples solution': The authors say iSamples will be 'collaborating with similar efforts globally' but they do not state/reference what these efforts are. The reader cannot infer what is meant here. Are similar efforts for identifying and indexing samples meant or is it similar efforts providing services for creating and assigning identifiers? It would be helpful to clarify this by stating the specific efforts with which collaboration will take place.
- In the last sentence of the text headed 'iSamples solution' the authors say with reference to figure 1 that to achieve the goals iSamples must advance standards and vocabularies across natural history domains. Was it intended to pick this domain specifically (which is generally taken to mean samples of plants and animals collected from the wild) or is the wider natural sciences domain what was really meant (i.e., samples of all biological (plants, animals, etc.) and non-biological (fossils, rocks, soil, etc.) materials occurring in the natural world)? I find this confusing at this point in reading the article although perhaps it becomes clearer later.
- In figure 1 the distinction between the blue (purple?) and purple (lilac?) dots is hard to make.
- With a 10-reference limit on commentary articles it is not possible to reference everything mentioned. Nevertheless, it might be worthwhile to consider replacing the One Health reference [10] with one to CARE instead e.g., http://doi.org/10.5334/dsj-2020-043 as this is a critical new social consideration of making sample based data more accessible. Interconnecting disciplines is already well-known for some years.
- In the technical description paragraph, the first sentence describing what iSamples Central is could be broken into two sentences after 'discovery and retrieval'.
- In the explanation of figure 2, the short-form words 'ID' and 'sync' are used when it would read better to spell out their long forms – identifier, synchronised.
- Change comma to full-stop immediately after the reference to figure 3 and begin a new sentence.
- Paragraph describing provenance. The last sentence would read better if it were broken after the phrase '…cannot always be inferred' and the paratheses around the example forming the latter part of the present sentence were removed.
- The screenshots forming parts of figure 3 would benefit from being taken from a larger screen so that the resolution can be improved at this PDF page scale.
- In the list of abbreviations, DataOne appears but it is not used in the main text.
- The selected references are appropriate. However, most of them are missing the year of publication and a DOI.

Are revisions requested?

Yes.

Are there ethical or competing interests?

No.

END.
