# Supplementary material for: Internet of Samples (iSamples): Toward an interdisciplinary cyberinfrastructure for material samples
Source: Gigascience. 2021 May 7;10(5):giab028. doi: 10.1093/gigascience/giab028 (PMC8103498; doi:10.1093/gigascience/giab028)
Supplement: giab028_Response_to_Reviewer_Comments_Original_Submission [file giab028_response_to_reviewer_comments_original_submission.pdf]

Author's Response To Reviewer Comments

Close

We are grateful to both reviewers for their careful consideration of this manuscript and below we provide point-by-point responses (bullet points) to their comments (in italics):

Reviewer #1:

Figure 3. … the explanation of the provenance (sample tree) relations among the things is correct but the assertions that the numbers in the sample tree are ARKs is incorrect. This explanation needs to be revised.

Figure 3: we have corrected the problems identified with the numbers in the sample tree

In figure 1 a distinction is made between iSamples digital objects and iSamples physical objects but no explanation is given of the difference. What is meant by the former?

We intend to eventually extend iSamples to digital objects, and the ID will be required for these as well. However, this paper simply describes our work for material samples, so we have simplified the language in figure 1 and its legend.

Also in figure 1, it is mentioned that facilitating community-driven metadata standards and adopting interdisciplinary metadata profiles will take place. These are social challenges. Later in the article, there is an admission that significant social challenges exist but not much is said about how the project aims to tackle these.

We intend to address these challenges through a Research Coordination Network "Sampling Nature". We have not cited this RCN directly, however, because the award is not yet finalized. Instead, we have revised the text to mention the importance of RCNs as a mechanism for engaging the community in the social aspects of cyberinfrastructure.

In the explanation of figure 2 the phrase 'identifier coordination' is used. What is meant by this?

Identifier coordination involves ensuring that newly minted identifiers are associated with minimal metadata and that such records are collated locally and globally. We have added this additional explanation to the Figure 2 caption.

It is said that iSamples Central index also stores links to related data and publications as well as the metadata about the samples. How these links will be captured and created is not explained.

We have added an explanation to the Figure 2 legend.

No mention is made… organization-specific (legacy) identifier types arising from historic collections ...How can these cases of non-unique identifiers be accommodated by iSamples?

We have updated the text in the Figure 2 legend to address this issue (briefly). A fuller response: While legacy non-unique identifier types are an important issue, iSamples is focused on the need for all new samples to follow best-practice in assigning unique identifiers and thus not to contribute to the scale of the problem going forward. Having said that, assurance of legacy record uniqueness can be achieved through namespacing (e.g., prefixing with an ARK shoulder) or minting of a new identifier. We plan to take advantage of the California Digital Library N2T (name to thing) resolver service to help manage legacy identifiers with an option to convert legacy identifiers into globally unique "compact identifiers". [https://doi.org/10.1038/sdata.2018.95] In either case, the original identifier is retained with a "sameAs" relation to ensure ongoing resolution, albeit with potentially multiple matches that require additional refinement. iSamples in-a-box services would allow legacy institutions without identifier best

practices to participate.

...how will machine/software-oriented rather than human-oriented processing of digital data about samples be catered for. The article does not discuss machine-actionability of sample data at all; although the authors acknowledge that further work beyond the present project will be required.

We very much agree that machine-actionability is a key issue and iSamples represents a first step by making sample data FAIRer, and as the reviewer points out, the FAIR principles are themselves aimed at machines. We added a sentence recognizing that sample cyberinfrastructure must strive to ease frictions of software (machines) interacting with the (meta)data.

Access and benefit sharing should be mentioned alongside CARE.

We have made this addition in the text

In the text headed ‘iSamples solution’: The authors say iSamples will be ‘collaborating with similar efforts globally’ but they do not state/reference what these efforts are. The reader cannot infer what is meant here. Are similar efforts for identifying and indexing samples meant or is it similar efforts providing services for creating and assigning identifiers? It would be helpful to clarify this by stating the specific efforts with which collaboration will take place.

We have revised the text to mention our work to align iSamples development with other international efforts.

In the last sentence of the text headed ‘iSamples solution’ the authors say with reference to figure 1 that to achieve the goals iSamples must advance standards and vocabularies across natural history domains. Was it intended to pick this domain specifically (which is generally taken to mean samples of plants and animals collected from the wild) or is the wider natural sciences domain what was really meant (i.e., samples of all biological (plants, animals, etc.) and non-biological (fossils, rocks, soil, etc.) materials occurring in the natural world)? I find this confusing at this point in reading the article although perhaps it becomes clearer later.

We have changed natural history to the natural sciences

In figure 1 the distinction between the blue (purple?) and purple (lilac?) dots is hard to make.

We have modified the colors. We also use an app that previews how colors look to people with different forms of color blindness, and modified the colors for maximum visibility across different vision profiles.

With a 10-reference limit on commentary articles it is not possible to reference everything mentioned. Nevertheless, it might be worthwhile to consider replacing the One Health reference [10] with one to CARE instead e.g., http://doi.org/10.5334/dsj-2020-043 as this is a critical new social consideration of making sample based data more accessible. Interconnecting disciplines is already well-known for some years.

In the technical description paragraph, the first sentence describing what iSamples Central is could be broken into two sentences after ‘discovery and retrieval’.

In the explanation of figure 2, the short-form words ‘ID’ and ‘sync’ are used when it would read better to spell out their long forms – identifier, synchronised.

Change comma to full-stop immediately after the reference to figure 3 and begin a new sentence.

Paragraph describing provenance. The last sentence would read better if it were broken after the phrase ‘…cannot always be inferred’ and the paratheses around the example forming the latter part of the present sentence were removed.

We have made these changes

The screenshots forming parts of figure 3 would benefit from being taken from a larger screen so that the resolution can be improved at this PDF page scale.

We have improved the resolution

In the list of abbreviations, DataOne appears but it is not used in the main text.

The selected references are appropriate. However, most of them are missing the year of publication and a DOI.

We have removed DataOne from the abbreviations and updated references where possible

Reviewer #2:

...there have been recent efforts in the US and Europe to develop the concept of the Extended Specimen (in the US), and the Digital Specimen (in Europe). These relate to the standards, metadata schema and persistent identifiers for Natural History specimens, so only part of the domain purported to be covered by iSample, but given that iSample is focusing on this sector first, I would have expected some mention of this initiative, such as the recent GBIF consultation on this topic.

We added these concepts as an example of how iSamples will connect to broader efforts and to domains beyond natural science (we cite public health as one example). Recognizing this we changed the subtitle of the section from 'Beyond Natural History' to 'Beyond iSamples'.

the text makes a few assumptions that are probably beyond most readers working outside the domain. For example, the Traditional Knowledge and Biocultural Labels initiative is part of a "Local Contexts" project, but there is no citation, or explanation of why this is important.

We agree that there would ideally be a citation here but given the tightly limited number of references allowed, we believe the initiatives/projects mentioned can be easily found through an internet search. We have revised the text to provide a fuller (if still necessarily very brief) explanation of why it is important.

There are a few minor corrections needed in the text. Specifically:

- "significant sociological challenges remain to unleashing [should be unleash] the full value".
- "Integration of CARE as well as FAIR principles" [CARE is not defined in the text, just the abbreviation].

We have made these corrections

- "iSamples will focus on the natural history sector - any sample where geolocation is of primary importance" [I don't understand the context of the geolocation reference or its specific link to natural history collections - its relevant to all collections at some level].

We have removed "any sample where geolocation is of primary importance"

Close
