# Supplementary material for: Internet of Samples (iSamples): Toward an interdisciplinary cyberinfrastructure for material samples
Source: Gigascience. 2021 May 7;10(5):giab028. doi: 10.1093/gigascience/giab028 (PMC8103498; doi:10.1093/gigascience/giab028)
Supplement: giab028_Reviewer_2_Report_Original_Submission — Vincent Smith -- 3/5/2021 Reviewed [file giab028_reviewer_2_report_original_submission.pdf]

Reviewer Report

Title: Internet of Samples (iSamples): Toward an Interdisciplinary Cyberinfrastructure for Material Samples

Version: Original Submission **Date:** 3/5/2021

Reviewer name: Vincent Smith

Reviewer Comments to Author:

This manuscript is essentially is a project description and a vision for the future of collection management systems, rather than describing something that is already constructed. The problem domain for institutional collection management systems (CMS) is well understood but poorly catered for, and iSamples proposes to deal with some of these weaknesses. This manuscript extends the vision for CMS in serval ways, through a new solution called iSamples. Firstly, iSamples abstracts the CMS problem domain across many collection sectors, it focuses on the assignment of persistent identifiers (specifically ISGN's) to metadata and specimens, and so far as I understand it, the proposed infrastructures works across metadata from multiple collections through a common data store providing a common infrastructure for data management and annotations across institutional collections. Having said this, I find some aspects of the manuscript quite confusing and much of the text has the appearance of being cut from a grant application. Its therefore very hard to test was is being said, mainly because the text isn't sufficiently detailed and what is plaid on is a high level construction plan.
Notwithstanding the fact that this is essentially a proposal, rather than a working system, there are a few other issues with the text. Firstly, there have been recent efforts in the US and Europe to develop the concept of the Extended Specimen (in the US), and the Digital Specimen (in Europe). These relate to the standards, metadata schema and persistent identifiers for Natural History specimens, so only part of the domain purported to be covered by iSample, but given that iSample is focusing on this sector first, I would have expected some mention of this initiative, such as the recent GBIF consultation on this topic (https://www.gbif.org/news/x7u6tmOhyfTE6rM3FQglK/community-consultation-converging-digital-specimens-and-extended-specimens). Secondly the text makes a few assumptions that are probably beyond most readers working outside the domain. For example, the Traditional Knowledge and Biocultural Labels initiative is part of a "Local Contexts" project, but there is no citation, or explanation of why this is important.
There are a few minor corrections needed in the text. Specifically:
- "significant sociological challenges remain to unleashing [should be unleash] the full value".
- "Integration of CARE as well as FAIR principles" [CARE is not defined in the text, just the abbreviation].
- "iSamples will focus on the natural history sector - any sample where geolocation is of primary importance" [I don't understand the context of the geolocation reference or its specific link to natural history collections - its relevant to all collections at some level].

Level of Interest

Please indicate how interesting you found the manuscript: Choose an item.

Quality of Written English

Please indicate the quality of language in the manuscript: Choose an item.

Declaration of Competing Interests

Please complete a declaration of competing interests, considering the following questions:

- Have you in the past five years received reimbursements, fees, funding, or salary from an organisation that may in any way gain or lose financially from the publication of this manuscript, either now or in the future?
- Do you hold any stocks or shares in an organisation that may in any way gain or lose financially from the publication of this manuscript, either now or in the future?
- Do you hold or are you currently applying for any patents relating to the content of the manuscript?
- Have you received reimbursements, fees, funding, or salary from an organization that holds or has applied for patents relating to the content of the manuscript?
- Do you have any other financial competing interests?
- Do you have any non-financial competing interests in relation to this paper?

If you can answer no to all of the above, write 'I declare that I have no competing interests' below. If your reply is yes to any, please give details below.

I declare that I have no competing interests

I agree to the open peer review policy of the journal. I understand that my name will be included on my report to the authors and, if the manuscript is accepted for publication, my named report including any attachments I upload will be posted on the website along with the authors' responses. I agree for my report to be made available under an Open Access Creative Commons CC-BY license (http://creativecommons.org/licenses/by/4.0/). I understand that any comments which I do not wish to be included in my named report can be included as confidential comments to the editors, which will not be published.

Choose an item.

To further support our reviewers, we have joined with Publons, where you can gain additional credit to further highlight your hard work (see: https://publons.com/journal/530/gigascience). On publication of this paper, your review will be automatically added to Publons, you can then choose whether or not to claim your Publons credit. I understand this statement.

Yes Choose an item.
